# Spirit of the English Periodicals, and Notices of English Medical Literature

**Published:** 1837-04-01

**Authors:** 


### Spirit of the British and American Periodicals, and Notices of British Medical Literature.

### Recent Researches in Anatomy.

### 1. Observations on the Thyroid Gland. By Mr. T. W. King.*

The thyroid gland is one of those structures, of which not only the use is unknown, but of which the mere anatomical structure is indifferently understood. Mr. King has investigated it with assiduity, if not success, and we shall present the results of his and of Sir Astley Cooper's inquiries. We shall condense the descriptions as much as we are able; but much compression cannot be usefully exerted on articles of this character.

A. *The Thyroid Gland made up of Lobes and Cells, containing a Fluid.*

"Many recent anatomists (says Mr. King) seem to have observed that the thyroid gland contains a fluid; a few have adverted to its lobular structure; and others to an opinion that its more minute organization is granular."

Mr. King has not quite done the "recent anatomists" justice. From aught that appears in the preceding passage, the reader might suppose that the cells of the thyroid gland had not been distinctly noticed, and that its fluid contents had not been pointed out. Yet the lobular arrangement, and the presence of fluid, are insisted on by Cloquet and transcribed by Quain, and Cruveilhier has particularly pointed out the cells.

"Cet organe (says Cruveilhier) présente tous les caractères anatomiques des glandes, et comme elles, il se sépare par la dissection en grains glanduleux; mais il y a entre ces grains glanduleux et ceux des glandes ordinaires cette différence, que, dans la glande thyroïde, les grains glanduleux communiquent les uns avec les autres, tandis qu'ils sont indépendans dans les glandes ordinaires. La communication des grains glanduleux entre eux est démontrée par les préparations suivantes. Si on pique la glande thyroïde à l'aide d'un tube à injection lymphatique rempli de mercure, on verra ce liquide se précipiter dans les cellules, qu'il distend, et au bout d'un certain temps tous les grains glanduleux sont injectés. Il est facile de s'assurer que le mercure n'est pas infiltré dans le tissu cellulaire, mais bien contenu dans le tissu même de la glande, au centre même des granulations. Le lobe droit ne communique pas avec le lobe gauche; mais toutes les granulations de chacun des lobes communiquent entre elles.

La glande thyroïde a donc une structure vésiculeuse: or nous avons vu que les grains glanduleux de toutes les glandes étaient spongieux et comme poreux, et que dans ces pores peuvent s'accumuler les produits de leur sécrétion.

La nature glanduleuse du corps thyroïde est encore démontrée:

1° Par l'humeur visqueuse, limpide, d'un œil jaunâtre dont il est pénétré chez quelques sujets, liqueur qu'on pourrait recueillir en assez grande quantité pour la soumettre à l'analyse:

2° Par la rétention de cette même matière dans un nombre plus ou moins considérable de vésicules, lorsque les orifices des communication de ces vésicules avec les vésicules voisines viennent à s'oblitérer."*

We do not quote these observations of Cruveilhier for the purpose of throwing cold water on what Mr. King has done, but, as he has volunteered an account of the information offered by other anatomists, it is only fair that that account should be correct. We now proceed to the description given by Mr. King, which he assures us, and we do not doubt him, is drawn from his own

* Guy's Hospital Reports, No. III.

* *Anatomie Descriptive. Par J. Cruveilhier. Tome Deuxième, p.* 689-90.

dissections, and supported by his preparations. The gland should be selected at an early adult period, fully developed, but without much condensation of the membranes. The examination will be facilitated, if the part be rendered moderately firm by immersion in alkohol.

Cellular membrane invests the gland, and forms a capsule for it. This sends prolongations into the interior, dividing the gland into laminated lobules, rather irregular in size and form, but about equal in extent to the little-finger nail, somewhat lengthened perpendicularly, and attached, for the most part inferiorly, by one end or edge. These lobules are very little susceptible of subdivision, and no where admit of complete separation without injury to the glandular texture. These lobules are rather larger internally than externally, and they are superimposed upon each other in a more or less concentric order.

The lobules are ultimately composed of cells, spherical or many-sided in form, minute in size, closely aggregated, and *without* perceptible apertures of communication.

" The proper contents of these cells are a fluid, which exists in widely-different quantities; and the cells are more or less visible, in proportion to the quantity of their contents. It is rare, however, to find them quite devoid of the secretion. An incision into the gland may scarcely yield a drop, and the cells may be actually invisible; yet, on immersing the part in water, the latter appears to combine with the secretion, and the cells are seen to become expanded in consequence. The cells must, indeed, become microscopic objects when emptied of their peculiar fluid; but even when no vestige of them appears to the naked eye, a portion of the gland, subjected to the action of alcohol or heat, will yield a vast number of minute grains of the consolidated secretion, which, as well as the containing cells, present very beautiful appearances under the microscope.

The most careful examination leaves little room for the supposition that the fluid belongs to any other part than the cells. It is of course impossible to define the largest size to which the cells may attain in the healthy or physiological condition; but it cannot be doubted, that a distention which materially exceeds the dimensions of a small pin's head must be morbid."

Mr. King attributes the granular appearance of the gland, insisted on by many anatomical observers, to the vascularity, under particular circumstances, of the parietes of the cells, and to an inconsiderable quantity of fluid in them. The thickness of those parietes diminishes as the vascularity decreases, and when the cells become distended.

The investing membranes of the cells are rendered very thin by the action of alkohol, whilst the contained fluid is consolidated almost without diminution.

B. *Analysis of the Fluid—Vessels and Nerves of the Gland.*

The fluid of the thyroid gland is certainly peculiar. In colour, it approaches the serum of the blood. Collected on the point of a knife (after incising the gland), it appears like weak gum, and is almost devoid of the ropiness of white of egg. Its bulk diminishes little when the substance is dried: at the same time, it becomes hard, somewhat darker, and less translucent. The fluid put into common rectified spirit seems only to lose a little water: it becomes solid, but not opake, and decreases but slightly. The same effects result in the cells when the gland is boiled for a quarter of an hour, and no apparent solution occurs. These are highly characteristic phenomena; although the coagulation, without opacity, may possibly be found to depend on very slight peculiarities of composition. The fluid, when mixed with water, seems to assume a turbidity with most of the ordinary tests for albumen; and, without dilution, may sometimes be rendered opake by a very weak nitric acid, as if it were that an alkaline influence is thus neutralized. The proper distinguishing characters are—its originally feebly mucilaginous form, and its subsequent bulkiness or massiveness when dried into a solid; and, again, its complete coagulation, without opacity, in alcohol, or when boiled, although the firmness thus produced is greater than that which results when the pure white of egg is operated on."

The small quantity of the fluid that can be satisfactorily collected, precludes, perhaps, exact analysis. Mr. H. R. Brett has examined some, and the result of his investigations would seem to amount to little more than this:— That one hundred grains of the dried gland yield five grains of fixed saline matter and oxide of iron:—

Alkaline chloride (most probably common salt);

Alkaline sulphate (mere traces);

Alkaline phosphate;

Phosphate of lime;

Oxide of iron;

Free alkali (probably potass), traces only.

The arteries of the thyroid gland are well known to be large and numerous. It admits of being minutely injected, though, like other organs in which injection and secretion are only occasional, its ordinary state is comparatively pale and bloodless. It is more vascular in infancy than at a later period of existence.

The veins are numerous and very capacious. The larger possess valves, yet the vessels can be injected from trunk to branch.

The absorbent vessels are many, and, comparatively, of large size. They pass out from, and cover all parts of the surface of the gland; form some junctions; and proceed, for the most part, into neighbouring absorbent glands. They sometimes appear to possess a rather less delicate structure than other lymphatics. Their valves are numerous: three or four pairs may occasionally be counted in a tube that is scarcely more than a quarter of an inch in length.

"The most important novel fact concerning the thyroid gland is doubtless this, that its absorbent vessels carry its peculiar secretion to the great veins of the body.* And the most simple and satisfactory method of demonstrating this fact is, to expel the contents of a healthy gland, by repeated and gentle compressions, into the lymphatics of the surface, and then to coagulate the fluid in these vessels. In this process, the delicate little lymphatics are first seen leaving the substance of the gland, and uniting, upon its surface, into trunks: and these, by the coagulation of their contents,* are rendered solid cords, with a very slight diminution of their capacity."

The contents of neighbouring absorbent vessels possess a smaller quantity of fibrinous and albuminous matter than the thyroid secretion does, and the latter must, therefore, be diluted in its passage to the venous system. There are many absorbent glands round the thyroid, and a few, of small size, appear to belong to it. One or two of them are impressed upon the lateral margins of the great lobes, posteriorly; one is often placed above the isthmus, and another in the mesian notch of the scutiform cartilage: some range beneath the under edge of the gland, and upon the trachea; and the smallest of them are but a very little larger than a pin's head.

The thyroideal nerves are inconsiderable.

*c. Relative Position of the Gland—its frequent Compression.*

The situation of the gland on the trachea and side of the larynx, covered by the sterno-hyoid and thyroid muscles, points out the frequent muscular compression and kneading it must be subject to. In the ascent and descent of the larynx in the act of deglutition, the gland must be additionally pressed on, from its form, and the disposition of the space in which it lies.

* "This has been indirectly surmised by Morgagni and others."

* "Upon collecting a little drop from one of these distended lymphatic ducts, and applying the test of alcohol, the solidity of the matter has remained as when within the gland, but its translucence has been less marked. I cannot say, however, that the small absorbent glands have had no share in this effect."

"There is a casual circumstance of deviation in the form of the thyroid gland, which may serve to strengthen the view of its alternating states of compression. It frequently happens that a slight flattened pillar of the gland ascends from the isthmus, on either side of the pomum Adami: it is seen, perhaps, rather more often on the left than the right side; and it rarely appears well formed on both.* It is invariably under the influence of the sterno-hyoid muscle: but it is now and then provided with its own muscle; which, descending from the os hyoides, covers the pillar, and is inserted about its base, near the isthmus; thus exerting an extra pressure upon the supplementary portion of gland."

D. *Uses of the Gland.*

Mr. King offers a sort of diagram of the various organs and tissues in the body, classified according to the character of the nutritive fluid they receive, and to that of the fluids and solids that they yield. The thyroid gland is placed in the same category as "bones, muscles, and appendages, coats of vessels, spleen, thyroid and thymus glands, and renal capsules." It might appear odd, that organs and textures so different from each other as the preceding, should admit of being packed into the same physiological basket; and we fear it must appear evident, that the uses of the thyroid gland are not materially elucidated by the classification. In fact, Mr. King offers us many conjectures, and hints at many possibilities, but he does, and that may be anticipated, little more. It would seem that the gland is chiefly destined for temporary, or rather occasional purposes, and its cellular structure and fluid secretion give a faint colour to the supposition, that it may constitute a sort of reservoir. Mr. King urges, and we would earnestly support his recommendation, a careful observation of the varying conditions of the gland, and an accurate investigation of both its natural and morbid states under different circumstances as regards the individual. He subjoins three cases, which really serve to shew so little, that we may dispense with any farther notice of them.

* "The production of the supernumerary portion, or pyramidal appendix to the isthmus, is found in various forms. Sometimes, in the absence of an isthmus, there is a double ascending flattened column; or, the isthmus being more or less complete, the pyramids are also developed: in either case, one or the other pyramid may be absent; one or the other, or both, may be imperfect in fulness or length, and occasionally surmounted by a small isolated spot of the same glandular structure. The occasional presence of a slip of a thyro-hyoid muscle, which is actually inserted into the gland (to raise it), or into the summit of a pyramid, is likewise worthy of note, in connexion with the view of muscular influence."

2. Notes on the Structure of the Thyroid Gland. By Sir Astley Cooper, Bart.

Sir Astley, between the years 1824 and 1830, made some inquiries into the structure of the thyroid gland. He then became aware of its cellular character, and of its fluid, a sample of which he sent to Mr. Babington, who pronounced it to be highly albuminous. Mr. King was not aware of what Sir Astley had made out, nor was Sir Astley, until the eleventh hour, acquainted with Mr. King's investigations. Thus, they tend to corroborate one another.

The most interesting portion of Sir Astley's communication relates to the comparative anatomy of the gland. We shall introduce what is stated on this subject both by Mr. King and by himself, in order that our readers may be put in possession of all that is precisely ascertained.

A *Thyroid Gland of the Cat.*

It contains a fluid, which is coagulated by the muriate of iron.

The structure of the thyroid gland is cellular.

In the rat, and the mole, a similar structure may be observed.—*Sir A. Cooper.*

B. *Thyroid Gland of the Dog.*

"After the removal of the two thyroid glands of a large living dog, they were found to contain a fluid, which was transparent, and coagulable by sulphuric acid."

"One gland is found on each side of the trachea. On the surface are numerous and large absorbents. When these are injected, besides absorbents, taking the course of the superior thyroideal artery, there is one to be observed on each side, descending to the junction of the jugular and subclavian veins behind the sternum. In 1824, when I put a ligature on the jugular vein and cellular membrane around it, a large absorbent vessel for the thyroid gland appeared."—*Sir A. Cooper.*

In the dog and cat, says Mr. King, the arrangement seems to be that of a number of small grains, more or less cubical (or polyhedral), closely packed into a flattened ovoid mass.

C. *Thyroid Glands of the Sheep.*

In the sheep there are two thyroid glands, generally united by a small intermediate portion. They are composed of cells, which are full of fluid. Mercury thrown into the arteries passes upon the cells, but does not easily enter them, for they are very minute. The veins admit of a more copious injection; and appear to communicate freely on the surface of the cells, where they form a network. The cells do not communicate with each other.

Sir Astley injected the thyroid gland of a sheep, by the arteries, with quicksilver. The artery, after entering the gland, divided into very small branches, which opened into little cells.

When examined under a microscope, the gland appeared, divided by a network, into cells; but each of these latter consisted of a great number of smaller cells.

The fluid removed from the gland of a sheep appeared, under the microscope, to contain a number of minute globules. It crystallized, like other animal fluids. It dried into a solid cake, like glue; which was, to a considerable amount, soluble in hot water. It coagulated with alcohol and sulphuric acid.—*Sir A. Cooper.*

In the ox, the fluid has characters resembling those of the sheep.

D. *Thyroid Glands of the Horse.*

"The horse has a thyroid gland on each side of the trachea, and each one receives two very large arteries. The structure is distinctly celullar. It is easy to fill the arteries and veins with injection, and to corrode them. When mercury was extravasated on the surface of one of these glands, it was immediately received into the absorbent vessels, and two lymphatic glands became filled."*—*Sir A. Cooper.*

E. *Thyroid Gland of the Pig.*

In the pig, the thyroid gland is placed on the fore part of the trachea, just above the sternum. It is large; and composed, internally, of numerous cells, which appear to have no communication with each other. The cells are distended with a fluid, which the gland secretes abundantly, and which may be procured by cutting into various parts of the organ.

Mercury was extravasated into the thyroid gland of a sow, four hours after the death of the animal. A few cells became filled; but the lymphatics soon conveyed the injection to an absorbent gland on the front of the trachea.—*Sir A. Cooper.*

Mr. King observes :—

"The gland of the pig projects in front only of the trachea, almost within the chest, and is closely covered with muscles. In some primates, as in man, it spreads laterally, but retains a con-

* "Five years ago I completely succeeded in injecting the thyroideal absorbents of the horse with coloured gelatine. Several vessels, escaping from the interior of the gland, passed on to some adjoining lymphatic glands; and finally formed a vessel, by the side of the trachea, as large as a small quill, proceeding from the gland to the veins. This preparation I have now sent to the Museum of Guy's Hospital."

siderable mesian isthmus. In the cat, the dog, the horse, and sheep, after birth (and mostly before), the isthmus fades into a mere film; but the lateral lobes are still subject to muscular compression, either upon an unyielding trachea, or the dilating œsophagus, which, in some instances, is so remarkable, as to constitute, perhaps, the greatest source of pressure.

In some creatures, the sterno-hyoid muscles seem to leave the surface of the trachea uncovered, in order to follow the glands towards the œsophagus: thus it is in the seal and porpoise. In others, again, the gland is inclined to surround the œsophagus posteriorly, so as to press between it and the vertebræ, as in the porcupine. The existence of a thyroid gland in birds has been doubted; yet in a region of goitre, the crows, as well as quadrupeds, are said to be subject to the disease. I find the bodies which seem to represent the thyroid in fowls to be placed within the chest, close on either side, against the distensible crop."

F. *Morbid Anatomy of the Thyroid Gland.*

The only observations of any moment offered on this head, are by Mr. King. He remarks:

"It is pretty obvious that nearly all the marked diseases of the thyroid gland consist in derangements of the natural cells; their partial or general atrophy; their dilatation, universally or in small numbers; the various alterations of the contents of the dilated cells, whether serous, bloody, or discoloured, albuminous, cartilaginous, bony, or puriform; the thickening, induration, or ossification of one or more of the cells subsequent to a morbid dilatation.—Other changes are less common. A general atrophy may be combined with more or less increase and induration of the cellular tissue of the gland, to which I have already adverted. I have also found the site of the gland occupied entirely by condensed cellular tissue, with the exception of two pea-sized portions of true gland situated on one side within this tissue. In this case, the peculiarities of the individual were fully sufficient to indicate the importance of the defective part."

Sir Astley promises to pursue the investigation in company with Mr. King. We wish them both the success that they deserve. It is no mean honour to the latter, to be associated with one who has done, and is doing, so much for minute anatomy as the worthy Baronet.

---

THE HISTORY OF AN UNUSUALLY FORMED PLACENTA, AND IMPERFECT FŒTUS, AND OF SIMILAR EXAMPLES OF MONSTROUS PRODUCTIONS. By DR. HODGKIN. With an Account of the Structure of the Placenta and Fœtus. By SIR ASTLEY COOPER, Bart.

We will first lay an account of the imperfect fœtus examined by Sir Astley before our readers, and then notice some of the interesting remarks of Dr. Hodgkin.

The mother of the fœtus, Mrs. D. aged 28, was attended by Mr. Randall, of Finsbury Square. She became pregnant in the beginning of February 1832, and felt herself as well as usual under similar circumstances; till between the 4th and 5th month, when she began to complain of a pain a little below the hypochondriac region on the right side; which increased in severity till the period of parturition, so as to prevent her walking to any distance, and to disable her from sleeping in the recumbent posture for full three months previous to her confinement. To secure any rest, it was necessary to prop the back with pillows.

These distressing symptoms were accompanied by a profuse discharge from the vagina, of a brownish-coloured fluid, great emaciation of the whole body, and extreme tumefaction of the legs.

During the first five months of pregnancy, the appetite was very bad: after this period, it became ravenous. She went her full time, suffering considerably to the very last. At two o'clock of Thursday the 18th of October, she was delivered of a fine healthy female child.

Within a quarter of an hour of its birth, severe pains returned; and, upon examination, it was evident that something more than the placenta was left behind, although no perfect presentation could be made out.

After excessive pain, attended with considerable loss of blood, and consequent exhaustion, the monster, now in the possession of Sir Astley Cooper, was protruded, in the horizontal direction. The placenta was thrown off about the usual time, and the uterus contracted firmly. On the 20th, Mrs. D. had an attack of inflammation at the spot which had been affected during pregnancy, but under appropriate treatment, she recovered. The living child has been subsequently healthy, but the pulsations of the heart are strong and full, and vibratory. We now proceed to the results of Sir Astley's examination of the placenta and of the imperfect fœtus.

"The placenta was composed of a single mass; but it was divided by its membranes into two cavities of unequal magnitude, the larger of which had contained a well-formed, and the latter an imperfect fœtus.

The larger child was surrounded by the usual membranes; and the smaller, and imperfect, was enveloped in similar and distinct coverings; but the membranes of each proceeded from the single placenta, and formed two cavities, perfectly distinct from each other, although they were seated upon the same placenta."

In the funis of the perfect child there were as usual two umbilical arteries and one umbilical vein, which diffused themselves in the placenta in the usual manner. The umbilical vein was rather larger than natural.

"The imperfect fœtus was contained in a cavity formed by a portion of the same placenta as that which contained the perfect; and was inclosed in membranes which surrounded it, and separated it from the larger fœtus; as the membranes were interposed between the two children, and also enveloped them.

The cavity was much more than sufficiently large for the imperfect fœtus, and probably had contained liquor amnii; but the membranes having been broken, the fluid had escaped. The base of this cavity was produced by a portion of the placenta, which was destined to the connexion of the imperfect fœtus; whilst the larger portion of the same placenta was connected with the perfect fœtus.

The membranes which surrounded the imperfect fœtus arose from the margin of the smaller portion of the placenta, and from a line which crossed its surface between the two children; and from this same line sprung the membranes of the perfect fœtus, as well as from the margin of the larger portion of the placenta. Thus the septum, or separation between the two children, was formed by the combined membranes of each; there being separate membranes, but only one placenta for the two children.

Near the centre of the cavity which contained the imperfect fœtus, a funis arose from that portion of the placenta; so that there was a funis to each fœtus. This funis of the imperfect child contained only one umbilical artery, and an umbilical vein. The umbilical artery divided into three considerable branches upon the smaller portion of the placenta, to which it was distributed.

The umbilical vein was of large size, and it received branches of veins from the smaller portion of the placenta, which corresponded with the branches of the umbilical arteries.

But now the great peculiarity of the mode of support and circulation of the second or imperfect fœtus is to be found in the structure of its placenta, and, as will be seen, in the formation of the fœtus itself. A large branch of the umbilical vein of the perfect fœtus passed upon the surface of the placenta, directly to the funis of the imperfect child, and thus established a free venous communication between the two children; whilst the other branches of the vein ramified upon the surface of each placenta. The umbilical arteries also of the perfect fœtus sent forth two large branches, but of unequal magnitude, to the smaller portion of the placenta, as well as many branches to the larger part of it; and the two arteries which passed to the smaller placenta formed only a single umbilical artery in the funis of the imperfect child: so that there was an arterial anastomosis between the two children, and a free communication between them was thus established.

The umbilical vein of each fœtus entered its navel in the usual manner; and the two umbilical arteries of the perfect child, and the single artery of the other, emerged from each umbilicus, to pass to the placenta."

*Structure of the Imperfect Fœtus.*

It was composed of a body and one leg, with a rudiment of a second thigh.

*Bones.* They consisted of a part of the dorsal vertebræ and those of the loins; and of three ribs, half the pelvis, the os femoris, patella, tibia, fibula, and those of the foot, which had only four toes, all on the right side.

*Muscles.* Nothing peculiar in them.

*Veins.* "The umbilical vein entered the navel at the usual place, and divided into a superior and an inferior branch. The superior became further divided into two vessels; one passing directly into the spine, to form the sinus venosus; the other splitting into two branches, which supplied the skin of the upper part of the body. The inferior branch sent an iliac vein on the right side, which divided into external and internal; and the latter sent forth the obturator vein. The iliac vein also sent a branch to the spine and to the integuments. Passing under Poupart's ligament, it received the femoral: proceeding further, the saphena vein, and a large cutaneous branch from the outer part of the thigh and leg, entered it; and these were well injected, from the umbilical vein even to the foot; shewing the absence of valvular office. The injection had indeed, in various parts of the body, passed so minutely, as to have reached the extreme ramifications; and had thence returned into the small branches of the arteries, where it was clearly visible. A small iliac vein passed from the umbilicus to the rudiment of the left thigh."

*Arteries.* The umbilical artery entered the fœtus by the side of the vein, and passed directly to the aorta, which was formed by two large branches at the upper part of the spine, and descended upon it, to form the two iliac arteries, the external and the internal, on the right side; after which it formed the femoral, under Poupart's ligament, and sent forth the usual branches to the thigh, leg, and foot.

*Nerves.* The anterior crural and saphenus nerve existed. The obturator nerve took its usual course. The spinal cord was large at its commencement in the dorsal region, opposite the superior rib. It descended to the pelvis, and formed the rudiment of the sciatic of the left side, and the sciatic and peroneal of the right, which were of large size and perfect formation. A small portion of sympathetic nerve was seen, with two of its ganglia, on the spine.

*Viscera.* The only viscus consisted of two inches of intestine, which was supplied with blood from the umbilical artery, as it entered; and a vein running to it was injected from the umbilical. It terminated in blind extremities.

There are some points connected with the structure, and the physiology of this fœtus and its placenta, which are not unworthy of attention.

A. When twins are formed, two placentæ are produced, which are, generally, not completely in contact, but connected by the membranes, which slightly adhere to each other: sometimes, however, the placentæ join, but without any vascular connexion: but still, in the practice of midwifery, the funis is secured, by way of precaution, to prevent hæmorrhage, lest such might be the formation.

When there are three children at a birth, the placentæ are generally distinct. When there is a quadruple birth, the same appears to be the case. Mr. Crosse, of Norwich, has, in his collection, two specimens tending to prove this. In one, the four placentæ lie two and two, but there are four dis-

Sir Astley goes on to remark that another hypothesis is possible. We may conjecture that the blood went *to* the imperfect fœtus by the umbilical *vein*, whence it passed into the aorta, and returned *from* the fœtus by the umbilical arteries. But this is the least probable supposition, as it supplies no circulating force, and it is opposed by the facts—*that* the umbilical artery of the imperfect, was plainly a continuation of that of the perfect fœtus—and *that* the part of the placenta belonging to the former, was evidently supplied by the umbilical arteries of the latter.

We may now advert to some of Dr. Hodgkin's remarks, and glance at another dissection of an acephalous fœtus by Sir Astley. The point on which we are busying ourselves at present, is the mode of circulation in fœtuses without hearts.

In almost all the cases of acephalous fœtus, the birth has *not* been single. Three cases of single birth have been recorded. In one of the three, related by Valisneri, the heart was present. In the other two, reported by Sulsmann and Donneaud, the heart was wanting, but we are ignorant of the precise condition of the vascular system. Deducting then these cases, we have a large proportion of imperfect fœtuses, which belonged to twin conceptions, and in which, therefore, the circulation of the monster *may* have essentially depended on that of the sound child.

In one case only, out of many of this description, has the communication between the umbilical cords been made the subject of inquiry. That case was published by Dr. John Clark, in the Philosophical Transactions of 1793. The dissection was made by Sir Benjamin Brodie.

The monster was voided after the delivery of a healthy child: it was enclosed in a distinct bag of membranes, composed of decidua, chorion, and amnion; and had a placenta belonging to it, the side of which was attached to the placenta of the healthy child. Before the internal structure was examined, the navel-string of the perfect fœtus was injected, from whence the injection readily passed through both placentæ, viz. that of itself and that of the monster, and then into the substance of the monster also, as appeared by the redness of the skin. The monster is stated to have contained a small portion of small intestine, which, as well as the other parts, was well injected: no other viscus was discovered. The vessels of the funis soon became, by their subdivision, too minute to be traced. The only bones discovered were, the os innominatum, an os femoris, with a tibia and fibula; but no cranium, spine, or sacrum. The monster is also *said* to have had neither brain, spinal marrow, nor nerves. The small portion of intestine had a peritoneum; and was attached, by a mesentery, to the os innominatum, where this bone is usually joined to the sacrum. In the funis, there appeared to be only an artery and a vein, which took their course towards the inner surface of the os innominatum: they passed backwards towards that part where the articulation of the os sacrum is generally found; whence they proceeded to the other side of the bone, and were soon minutely subdivided.

Dr. Hodgkin has collected and relates the following particulars, in reference to the distribution of the vessels in some recorded cases of monsters.

"In the case which Poujol examined, and in which he found no arteries, the umbilical vein opened into the vena cava, a little below the kidneys: the vena cava ascended along the spine to the chest, where it divided into two main branches, which again minutely subdivided. In Méry's case, the umbilical vein furnished branches to the intestines. In Gourraigne's case, corresponding branches of the umbilical vein and arteries were distributed over the fœtus, which appeared to want the vena cava and aorta, as well as the heart. In Cooper's fœtus, the umbilical vein, on entering the abdomen, divided into an ascending and descending branch; whilst an artery, analogous to the aorta, continued along the spine, and gave off the umbilical arteries. In one of Meckel's fœtuses, there was a large umbilical vein, which sent branches to the kidney, the intestine, and lower extremities: there was no vena portæ. In the 4th example observed by Tiedemann, the vascular system consisted solely of a single umbilical artery and vein."

Sir Astley describes another imperfect fœtus—a twin.

The head, neck, and part of the thorax were represented by a large cutaneous pouch. The remainder consisted of an abdomen, pelvis, and two lower extremities. The child was a female, and the external organs of generation were perfect.

*Vessels.* The funis contained an umbilical artery and an umbilical vein. The latter divided into two branches—one formed the sinus venosus in the spinal canal, the other the renal, capsular, mesenteric and femoral veins, with their branches. There was an aorta which supplied the existing parts, much as usual. From its iliac branch, on the left side, proceeded the single umbilical artery, to which we have already alluded. There was no heart. The only communication of the aorta with the veins was by means of the extreme branches of each.

*Nerves.* These were well developed. There was a spinal cord, which began at the second dorsal vertebra. There were sciatic, peroneal, anterior crural, and obturator nerves. The great sympathetic was very distinct on the left side of the spine. It presented six ganglia, and sent some nerves to the aorta, in the usual course of the aortic plexus.

The viscera which existed were, a part of the intestinum ilium, the cæcum with its appendix, the colon, and the rectum, connected to the spine by a mesentery and mesocolon. But the parts which chiefly occupied the cavity of the abdomen were, the kidneys and capsulæ renales, which were of great size, in comparison with other parts.

In the pelvis, the bladder, the uterus, two distinct ovaria, and Fallopian tubes, and the rectum, were all complete; and in the bladder, the ureters, which were of large size, terminated as in a perfect fœtus.

We have thus given a correct account of the principal part of this lengthened paper. It is of no mean physiological interest, for the first dissection made by Sir Astley has established, what was previously mere surmise, that the heart of the perfect fœtus may form the great circulating organ of the imperfect one. The establishment of a fact is always of importance. Men will now direct their attention to the point, and we shall have more accurate observations than we have had.

---

On the Brain of the Negro, compared with that of the European and of the Ourang-Outang. By F. Tiedemann, M.D.

"It has long been the prevailing opinion among naturalists, that the negro race is inferior, both in organization and intellectual powers, to the European; and that, in all the points of difference, it exhibits an approach to the monkey tribes. The object of the present paper is to institute a rigid inquiry into the validity of this opinion. The author has, for this purpose, examined an immense number of brains of persons of different sexes, of various ages, and belonging to different varieties of the human race, both by ascertaining their exact weight, and also by accurate admeasurement of the capacity of the cavity of the cranium, and has arrived at the following conclusions:—

The weight of the brain of an adult male European varies from 3 lbs. 3 ozs. to 4 lbs. 11 ozs. troy weight—that of the female weighs, on an average, from four to eight ozs. less than that of the male. The brain usually attains its full dimensions at the age of 7 or 8; and decreases in size in old age. At the time of birth, the brain bears a larger proportion to the size of the body than at any subsequent period of life, being then as 1-6th of the whole weight—at 2 years of age, it is 1-14th—at 3, 1-18th—at 15, 1-24th; and in the adult period, that is, from the age of 20 to that of 70, it is generally within the limits of 1-35th and 1-45th. In the case of adults, however, this proportion is much regulated by the condition of the body as to corpulence; being, in thin persons, from 1-22d to 1-27th, and in fat persons, often only 1-50th, or even 1-100th of the total weight of the body. The brain has been found to be particularly large in some individuals possessed of extraordinary mental capacity. No perceptible difference exists, either in the average weight or the average size of the brain of the negro and of the European: and the nerves are not larger, relatively to the size of the brain, in the former than in the latter. In the external form of the brain of the negro, a very slight difference only can be traced from that of the European: but there is absolutely no difference whatsoever in its internal structure, nor does the negro brain exhibit any greater resemblance to that of the ourang-outang than the brain of the European, excepting, perhaps, in the more symmetrical disposition of its convolutions.

Many of the results which the author has thus deduced from his researches, are at variance with the received opinions relative to the presumed inferiority of the negro structure, both in the conformation and relative dimensions of the brain; and he ascribes the erroneous notions which have been hitherto entertained on these subjects chiefly to prejudice, created by the circumstance, that the facial angle in the negro is smaller than in the European, and consequently makes, in this respect, an approach to that of the ape, in which it is still farther diminished. The author denies that there is any innate difference in the intellectual faculties of these two varieties of the human species; and maintains that the apparent inferiority of the negro is altogether the result of the demoralizing influence of slavery, and of the long-continued oppression and cruelty which have been exercised towards this unhappy portion of mankind, by their more early civilized, and, consequently, more successful competitors for the dominion of the world."*

We have inserted the preceding extract from a paper by Tiedemann. It is contained in a report of papers read before the Royal Society, in 1836. If the observations of the celebrated au-

---

* Athenæum, Dec. 31st, 1836.

thor be correct, the negro will have been long and grossly calumniated, and he will be proved to possess as much brains, at all events, if not as much intellect, as his European master. It is singular, however, that the various modes adopted of measuring the cranium should have been attended with such inaccurate results. The "facial angle" of Camper, and the "occipital angle" of Daubenton, are well known to be in many respects fallacious; but the vertical section of the cranium and face, recommended and adopted by Cuvier, appears to offer a less exceptionable method of determining their respective dimensions.

If different parts of the encephalon have different intellectual functions, as it is most likely that they have, it is obvious that the mere gross weight of the *whole* brain is not conclusive in determining the relative intellectual capacities of the different races of mankind. And, whether there be really different intellectual organs or not, it is certain that many portions of the brain are concerned in giving origins to nerves, and in receiving or originating the sensitive and motor columns of the spinal marrow. Much of the encephalon is, in short, appropriated to the animal functions, which, if not independent of the intellectual, must be distinguished from them—another source of error, sticking to conclusions drawn from the weight of the entire mass.

Yet, with all these reservations and allowances, M. Tiedemann's observations will take most physiologists by surprise, and fairly deserve farther consideration.

---

### Recent Investigations into the Composition of the Brain and Spinal Cord.

The subject which, of all others, engrosses the attention of the anatomists and physiologists of the day, is the nervous system. At this very moment, the most ingenious and the most industrious of the profession are exploring its history, its growth, its composition, and its functions, with singular earnestness—let us hope, with equal success. The present Number of this Journal is evidence of the intensity of interest displayed by the inquirers, and of the corresponding interest which, we feel assured, is entertained by the profession. Anxious to lend our assistance to the extension of knowledge, of so high and so important a character, we have devoted many pages to generalizing the facts which have been more or less completely elicited, and giving publicity to the laudable exertions of several of our countrymen.

We noticed in our last Number a work on the Nervous System, by Mr. Solly, Lecturer on Anatomy at St. Thomas's Hospital. We have not the pleasure of a personal acquaintance with that gentleman, but a perusal of his work has left a very favourable impression on our minds. It has shewn him in the light of a zealous anatomist, and of a man of a candid and liberal mind. We feel great satisfaction in laying before our readers the result of his dissection of the medulla oblongata.

It is, perhaps, hardly necessary to observe that the medulla oblongata is the bulbous upper extremity of the spinal marrow—that it displays in front the corpora pyramidalia, which decussate, more or less completely, in the anterior longitudinal groove which separates them—that, on the sides, are the corpora olivaria—and, posteriorly, are the posterior pyramids, or corpora restiformia.

If the fibres of these several bodies, themselves continuations of the several columns of the cord, be traced upwards, they are well known to expand into the cerebrum and cerebellum.

The anterior pyramids, passing through the pons, continued in the crura cerebri, traverse the corpora striata, and expand into the cerebral hemispheres. They are described, too, as traversing the thalami.

The corpora olivaria also penetrate the pons, the crura cerebri, the optic thalami, and are continued into the convolutions of the summit of the hemisphere, as well as into those of the posterior lobe. The corpora olivaria have also been observed to contribute their medullary investment to the tubercula quadrigemina, and to form the valve of Vieussens.

The corpora restiformia, constituting the inferior peduncles of the cerebellum, diverge into its lobes, and are supposed to be its only media of connexion with the cord.

The preceding are the generally-received opinions and descriptions, and we have given this summary sketch of them, in order that our readers may fully understand what Mr. Solly has done. His investigations relate to the destination of the anterior pyramids, and to their connexion with the cerebellum. His discoveries were communicated to the Royal Society in a paper, which was read in May, 1836, and Mr. Mayo and Mr. Owen, who were deputed by that Society to report on it, have admitted the accuracy of the facts that it contains. The preparations establishing the succeeding statements, are deposited in the museum of the College of Surgeons. Let us now listen to Mr. Solly.

The anterior pyramids communicate with the cerebellum by two sets of fibres—one superficial and one deep.

"The *superficial* may again be divided into two sets: the first cross the surface of the cord immediately below the corpus olivare, and may generally be seen without dissection; they are more distinct in the sheep, bullock, and horse than in man, in whom they form a very thin layer emanating from the corpora pyramidalia, and I have no doubt that they actually decussate with their fellows of the opposite side, forming, in fact, part of the apparatus of decussation, though I have not yet positively ascertained the fact.

The *second* of the superficial set of fibres take the same direction; only, instead of crossing the cord immediately below the corpus olivare, they run to the inner side of the corpus olivare, and then ascending to the cerebellum, they form the outer part of the corpus restiforme.

The *deep* set of fibres from the antero-lateral columns to the cerebellum, are the most posterior of the whole mass of fibres composing this portion of the spinal cord. They are separated from the posterior columns by the posterior fissure, from which the posterior roots of the spinal nerves emerge; this fissure they cross in their passage to the cerebellum, obliterating it entirely.

Thus it will be perceived that one portion of the antero-lateral columns—for there is yet another portion of these columns to be described—on reaching to within a small distance of the corpus olivare, splits into three sets of fibres, one, and the most anterior, of which passes through the pons Varolii, as will be described presently, and may be designated the *cerebral* fibres of the anterior columns; a second set, which may be entitled the *superficial cerebellar* fibres of the anterior columns, passing over the surface of the medulla oblongata are usually seen without dissection. Rolando describes the superficial cerebellar fibres of the anterior columns, those which are seen without dissection (the processus arciformes of Santorini), under the name of 'filamenti arciformi,' saying, 'I believe that I ought to give such a name to numerous filaments which are seen to issue from the transverse fibres of the annular protuberance precisely at the same spot where the anterior cords penetrate into its centre. The filamenti arciformi nevertheless descend and partly cover the abovementioned cords, expanding on the corpora olivaria, and extending even to the median fissure, by which they remain separated from each other. Such a disposition is constantly observable in quadrupeds, in which the said filaments are extremely distinct, although no mention has hitherto been made of them.' Rolando does not, however, trace them, as he might have done, to the cerebellum, instead of describing them as descending from the pons Varolii. The third or *deep cerebellar* fibres of the anterior columns, proceeding in company with those of the posterior columns, form about a fourth part of the whole diameter of the restiform bodies."*

* Solly on the Human Brain, &c. 12mo, 1836.

The physiological inferences that hang on these facts shall be stated in Mr. Solly's own words :—

"The fibres just described as connecting the antero-lateral columns of the cord with the cerebellum are peculiarly interesting when viewed in relation to the functions of the cerebellum. For although it is true that its functions have not yet been clearly ascertained, the experiments of Flourens, Bouillaud, Magendie, and others, and the numerous cases on record in which disease of the cerebellum has been followed by paralysis, all tend to prove that the cerebellum is in some way or other connected with the regulation of muscular action, most probably, as before hinted at, that it has the power of combining the action of individual muscles so as to effect an harmonious result, such as is necessary to enable us to stand, walk, &c. Even Broussais in his lecture on Phrenology, published in the Lancet, July 30th, 1836, acknowledges that the cerebellum is an instrument connected in some degree with the combined action of the muscles, though merely in relation to the act of copulation. Their presence also proves the weakness of Mr. Walker's theory of the function of the posterior columns as derived from the supposed fact that the posterior columns alone are connected with the cerebellum.

The circumstance is also at variance with the opinions of M. Foville, who reasons that the cerebellum must be concerned in the phenomena of sensation because the posterior columns are alone connected with it; while Dr. Prichard in his treatise on Insanity and other disorders of the mind, p. 482, after speaking of Foville's doctrines and their foundation upon what he considers an established fact in anatomy, says with the usual caution of such a highly talented observer: 'In the present state of these researches it would be a rash attempt to draw inferences with any degree of confidence; but I may be allowed to remark that the general bearing of facts seems to direct towards the conclusion that the two great organs inclosed within the skulls of vertebrated animals belong respectively to the two principal functions of animal life, which are, first, sensation, conscious perception, and the physical phenomena related to intelligence; and secondly, those of voluntary motion. This however can only be considered as a probable opinion. Such it has long been thought by many physiologists; and though the grounds on which this conclusion rests appear to be more secure than they formerly were, the proof is still defective."

---

### ANATOMICAL REMARKS ON HUNCHBACKS.*

Dr. Stern has published in Muller's Archiv. some remarks on the structure of hunchbacks, from which we abstract one or two particulars.

There is a sort of family likeness in all hunchbacks, arising from a similarity in complexion, and in form of head, as well as from a general care-worn and superannuated appearance of the face. The dimensions of the cranium and of the face are altered from their ordinary relation to each other—the former equalling in size that of a well grown adult, while the bones of the latter remain undeveloped and small, as in childhood. Hence, in their physiognomy we see the bizarre and unpleasing combination of old age, and wisdom, and childhood, and simplicity. In the form of the lower jaw, in the great size of the mouth, in the compressed flatness of the lips, and in the sharp elongated nose, we recognize a striking likeness between all hunchbacks.

The limbs are fashioned to serve a trunk of larger proportions than that to which they actually belong. Yet even here the growth is unnatural, for the different bones of the extremities do not bear their due ratio to each other. Thus the femur is somewhat shorter than it ought to be, while the bones of the foot are excessively large. Of all the bones, the humerus is proportionally the

---

* Dub. Jour. Sept. 1836.

longest, and to this is owing the great comparative length of the upper extremities in persons thus deformed.

---

Morbid Anatomy of Typhus Fever in Paris, Geneva, Dublin, Glasgow, Liverpool, Manchester, and London. By Dr. Lombard, of Geneva.*

The following facts observed by Dr. Lombard, are calculated to give rise to useful reflection in the mind of the pathologist and of the practical physician.

In Paris and Geneva Dr. Lombard has either made or seen a great number of dissections of patients dead of typhus fever. In all of them, without exception, he has found enlargement of the aggregated follicular glands in the lower third of the ileum, attended with ulcerations of the mucous membrane. So constant is this post-mortem appearance on the Continent, that it is looked on as an essential element in fever.

With this experience, Dr. Lombard went to Glasgow. The fever cases there were similar, almost identical, in character, to those he had witnessed abroad. A patient died, and he entertained no doubt of discovering follicular disease. There was none.

When Dr. Lombard arrived in Dublin, he again had opportunities of examining the bodies of fever patients. He inspected two, but found no follicular disease. He was informed by the physicians of Glasgow, that such lesions occur in about one-third of the bodies examined after death. The Dublin physicians told him that the alterations are even less frequent in that city, particularly during the present epidemic, which has preserved nearly the same characters for the last eighteen months. In former epidemics those changes appear to have been more common.

In Liverpool, where there were many cases of fever, Dr. Lombard found them similar in character to those of Dublin. The morbid appearances were similar too. Serous effusion in the brain, meningitis, pneumonia, and occasionally some injection of the mucous coats of the intestines, and occasionally also ulcerations of the ileum and cæcum, varying in frequency with the different seasons, composed the catalogue.

In Manchester, the total number of typhous patients received into the fever hospital in the year did not exceed three hundred and thirty two, a proportion far inferior to that observed in Liverpool and Glasgow. Dr. Lombard was informed that it was by no means constant for intestinal ulcerations to be found in the bodies of the dead.

In Birmingham, the fever cases are not numerous, but Dr. Lombard learnt that ulcerations of the lower part of the ileum were invariably found.

In London, the annual number of admissions into the Fever Hospital has been diminishing for the last three years. In 1835 the number of typhus cases was 250. Serous effusion in the ventricles and other morbid appearances of the brain, are more frequently to be met with, than the abdominal complications which in the shape of ulceration are not to be found in more than one fourth of the cases, according to Dr. Tweedie's researches; and this proportion of ulcerations in the lower part of the ileum varies with different seasons, being much greater in Autumn than at any other period of the year. The continued fever of London is very different now from what it was some years since; it was then highly inflammatory, and local and general blood-letting could not then be neglected; while now the symptoms of depression and weakness have taken the lead, and so low is the present fever that wine, camphor, and spirit of Mindererus are to be given in almost every case.

Thus, we have seen that in Birmingham only are intestinal ulcerations said to be constant. Dr. Lombard proposes an ingenious hypothesis, to account for the discrepancy in the morbid anatomy of fever here and on the Continent. But it is too unsatisfactory to induce

* Dublin Journ. Sept. 1836.

us to go into it. On one point, however, we find it difficult to agree with Dr. Lombard; and that point is no trivial one. We are not sure that follicular enlargements or intestinal ulcerations *do* attend all typhous fevers abroad. In support of this observation we may refer to M. Andral himself, who classifies "ataxo-dynamic," or typhous fevers thus:—1. Those in which the follicles are altered; 2. Those in which there is simple inflammation of the gastro-intestinal mucous membrane; and, 3. Those in which *no* lesion of the digestive tube exists.

M. Louis, indeed, has chosen to restrict the term typhus, to cases of the first class. But it may be questioned whether this is not, even in France, an arbitrary nosological definition, rather than an accurate expression of the fact.

---

### Dr. C. J. B. Williams on Percussion.*

Dr. Williams, an ingenious man and a zealous cultivator of auscultation and percussion, has published some remarks upon the latter subject, which are not undeserving of attention. His objects are, first, to explain the cause of sound on percussing the walls of the thorax; secondly, to deduce from such an explanation, some useful rules of practice.

1. He concludes that the sound obtained upon percussion, depends not on the vibrations of the air within, but on those of the solids themselves. His reasons are four.

A. If the sound depended on the quantity of air contained in the thorax, its tone should be deepened by the increase of that quantity. But a full inspiration raises the tone of the sound upon percussion.

B. If the sound depended on the air within the chest, it should be changed by the closure of the glottis. But, cæteris paribus, it is not.

C. "The sonorous vibrations of air contained in cavities may be of two kinds. One depends on any impulse communicated to the mouth of the cavity, and resisted by the body of air within, acting in the manner of a bottle, or of a tube closed at one end. This requires that the cavity should communicate with the external air, which we have seen is not necessary to the pectoral sound. The second kind of sound produced in air-filled cavities, is that resulting from the successive reflections of any sonorous impulse from side to side of the cavity; which reflections becoming regular vibrations, constitute a sound of a determinate character. This constitutes the prolonged reverberations heard in empty casks, the tinkling echo of the interior of bottles, cups, &c., the phenomena of metallic tinkling and amphoric resonance heard in various smooth air-filled cavities of the animal body.† But this cannot constitute the sound of pectoral percussion; for in a cavity of the size of the chest, instead of being drum-like, it would be tinkling, as it actually is in pneumo-thorax. Further, the pulmonary tissue, as it prevents the transmission of the voice from the bronchi to the surface, except at points where these are quite superficial, so much more completely will it prevent the transmission and reflection of sonorous vibrations to-and-fro, across the whole interior of the chest."

D. The lung is really a sort of air-filled sponge—a tissue adapted to arrest the vibrations of the air.

For these reasons Dr. Williams is of opinion, and we coincide with him, that the sound on percussion is really due to the vibrations of the parietes, and is affected by their tension, as well

---

* Med. Gaz. Jan. 21, 1837.

† "This tinkling resonance is interesting, as it gives rise to several instructive phenomena of auscultation. It may be illustrated by holding the mouth of an India-rubber bottle to the ear, and striking the side of the bottle: each stroke is attended, or rather followed, by a metallic clink.—See Pathology and Diagnosis of Diseases of the Chest, 3d edit. p. 115."

as by the resistance offered to them, by surrounding or subjacent parts. Those parietes may be compared, not *quite* accurately it is true, to the parchment or the lateral walls of the drum, whilst the cavity of the chest and its content represent *its* interior. As the parietes of the thorax are not uniform in structure, consisting of bone or cartilage, with interspaces of membrane and of muscle, and irregular external paddings of muscle, fat, or mammary gland, it is obvious that their tension is unequal, and it is clear that an advantage must ensue from the use of the pleximeter, or of the finger of the percussor, either of which tends to correct, on the nonce, that inequality. This view of the sounds upon percussion appears to us so much more consonant with all we know, than the hypothesis that the sound depends on the actual vibrations of the pulmonary air itself, that we need not go farther into the argument. Occasionally, as in pneumo-thorax, that air *does* vibrate, and then another sort of sound ensues—the metallic tinkling. So says our friend Dr. Williams; but we think that this is dubious. In pure pneumo-thorax, we have not necessarily the metallic tinkling, but an unnatural and clear resonance. This is not surprising. The parietes of the thorax are in a state of tension, and the large quantity of air in the pleura, is highly favourable to their perfect vibration. And in this case, as in the drum, a double vibration and a double source of sound exists—the contained air *and* the containing wall vibrate. The metallic tinkling is usually the consequence of liquid and air in the pleural cavity. The slightest agitation of the liquid readily occasions the sound in question, though the latter *may* be independent of it.

2. We pass to the application of the preceding theoretical conclusions. The object is to percuss to the best effect.

The first precaution to be observed is, that the spot percussed shall be in such a state of tension that it may vibrate; and if this be not the natural condition of the part, a degree of pressure, with the finger or pleximeter, will give this condition to it. The percussion should, of course, be perpendicular to the surface, and corresponding points of the two sides of the chest should of course, also, be selected for comparison. The force of the stroke arrests the attention of Dr. Williams.

According to that force must be the extent of the vibration—according to the latter must the parts *in* the thorax be affected. Suppose a thin stratum of serum, between the surface of the lung and the thoracic wall. Gentle percussion would produce a dull sound, because the fluid would modify the weak vibration. But strong percussion, occasioning forcible vibrations, their sound would be affected not simply by the superficial fluid, but by the lung subjacent to it. We don't know that it is necessary to follow out this principle in detail—a principle which serves to explain some otherwise obscure facts relating to percussion.

We have had some small experience in auscultation and percussion, and a fair share of that experience is in private practice on the better classes of society. The science of percussion is one thing—what actual circumstances will permit is another. We have little hesitation in saying, that the nice diagnoses seen upon paper, will in general *not* be obtained from the persons of ladies and gentlemen. Here and there a case may occur, a case of much obscurity, where a very minute examination is permitted. But, in general, both tact and reason dictate a more superficial investigation, and when the general symptoms and the physical signs lead to a *general* conclusion, to an approximation to the exact truth, we must be usually content. Yet nothing is more true than that minute knowledge is requisite for the formation of an accurate general opinion. This results from a quick appreciation of small circumstances, and how can they be appreciated unless we are familiar with them? The necessary superficial character of the ordinary investigations in private practice, are a stimulus to industry, not a damper on it—an additional motive for exertion, not a premium upon indolence. If a man has to fight with only one hand, he ought to be able to fight well.

### RECENT RESEARCHES ON THE SENSE OF TASTE.

### OBSERVATIONS ON THE FUNCTIONS OF MECKEL'S GANGLION, OF THE VIDIAN NERVE, OF THE PALATINE NERVES, OF THE GLOSSO-PHARYNGEAL, AND OF THE GUSTATORY.

1. *Experiments of Dr. Alcock on the Glosso-pharyngeal, the Gustatory, Meckel's Ganglion, and the Naso-palatine Nerves.**

2. *Remarks on Dr. Alcock's Experiments. By D. Noble, Esq.*†

3. *On the Functions of Meckel's Ganglion, and of the Glosso-Pharyngeal Nerve. By M. W. Hilles, Esq.*‡

We have lately had an opportunity of observing the contradictory nature of experiments. The mere experimenter may sometimes wilfully deceive, will constantly be liable to self-deception. Unless experiments are superintended, if not directed, by reasoning, they will frequently lead to the grossest fallacies, and seldom conduce to the establishment of truth.

The tongue is the organ of taste. Every body is aware that taste is most perfect in the anterior portion of that organ. If you wish to appreciate the sapid qualities of objects, you apply to them the tip of the tongue—if you wish to avoid tasting them, you thrust them far back. Who does not know that a colocynth pill, if placed on the back of the dorsum of the tongue, may be swallowed without the swallower learning its bitterness?

But taste is probably not a simple sense—at least what is generally called taste is not. It is partly made up of touch, partly of smell, and partly of an appreciation of the qualities of bodies, which resides in the palate, in the nares, and perhaps the pharynx. If the nose is held when a black draught is gulped, some of its nastiness is lost. We do not *taste* that portion of its flavor, which resides in its odour, or, possibly, in other qualities.

The back of the tongue and the fauces are deficient in the pure sense of taste.* That seems certain. But they have a sense which the anterior portion of the tongue has not—an exquisite sensibility, which, on the contact of a foreign body, instantly draws on a convulsive action of the muscles engaged in the act of deglutition, and especially those of the palate and the pharynx. If, in looking into the mouth, the spatula be placed a little too far back, we see repeated gulps, in other words successive series of contractions of the levators of the palate and the pharynx, of the constrictors of the latter, and of the elevating muscles of the larynx. All this from a touch of the mucous membrane, the light touch of a feather, or the heavy one of a spatula.

When we investigate the anatomy of these parts, we find,

1. *That* the anterior portion of the tongue, which enjoys taste *and* perfect touch, is similar in structure to those portions of skin in which the latter sense is most developed. A highly vascular cutis, is crowded with papillæ, themselves highly vascular, and gifted with the power of erection.

2. *That* the mucous membrane of the dorsum of the tongue, of the soft palate, and pharynx, no longer displays the same character of papillæ, but has numerous mucous follicles and glandules.

3. *That* a nerve, the hypoglossal, is distributed to the muscles of the tongue, and forms with the "gustatory," a plexus, by which those muscles are supplied.

4. *That*, the "gustatory," a division of a nerve proved to be sensitive, the

---

* Med. Gaz. Nov. 26, 1836.

† Med. Gaz. Dec. 17, 1836.

‡ Med. Gaz. Jan. 14, 1837.

* We do not mean that they are absolutely incapable of taste, but simply that it is not so perfect as in the tongue. The exact proportions of the sense possessed by different parts of the mouth and fauces, are not ascertained. Probably, they vary much in different animals, and experiments on one species are not conclusive with regard to others.

fifth, is lost in that part of the tongue where taste and touch in perfection reside, its anterior and papillary portion.

5. *That* another nerve, the glosso-pharyngeal, supplies the mucous membrane of the base of the tongue, *and* the muscles of the pharynx.

6. *That* other nerves, the palatine, and naso-palatine branches of the fifth, and connected with the ganglion of Meckel, supply the mucous membrane of the hard and soft palate.

If anatomy tells us any thing, if reasoning is not utterly fallacious, both conspire to shew—

1. *That*, as the gustatory supplies that portion of the tongue where taste is in its perfection, it must be *the* nerve of taste.

2. *That*, as the hypoglossal supplies the muscles of the tongue; it must be *the* motor nerve; and as the motions of the tongue are subservient to taste, or are regulated by it and touch, so the motor nerve and the sensitive one form, as usual, a plexus.

3. *That*, as the glosso-pharyngeal, supplies the mucous membrane, where a special sensibility resides, and the muscles of a tube which that sensibility remarkably affects and governs, it must be *the* nerve, which gives the endowments of sensation and of motion, which makes the base of the tongue feel as it does feel, and the pharynx contract as it does contract, in accordance with that feeling.

4. *That* if any sense analogous to taste, and probably there is such, does reside at the basis of the tongue, the glosso-pharyngeal will confer it.

The structure of the tongue so similar to that of the skin, the character of its nerves, the gustatory and glosso-pharyngeal, both so analogous to nerves of sensation (the latter being one of motion too)—contribute to confirm, what has always been more or less believed, that touch and taste are not separated by a very bold partition, and that the basis of the tongue may without difficulty be supposed to possess a tertium quid, a less perfect degree of those senses, both of taste and touch, which reside in the anterior portion of the organ.

When M. Panizza contended that the glosso-pharyngeal was *the* nerve of taste, we could do no more than shrug our shoulders, and wonder how a physiologist could believe that a nerve enjoyed, par excellence, a faculty, which did not, par excellence, reside where that nerve was. This was the obvious objection of reason, and events have proved how little in this case, as in many others, experiment is to be trusted, when plain reason is against it.

We shall now present a brief, but a sufficient account of Dr. Alcock's experiments, and of Mr. Hilles' suggestions.

### A. *Division of the Gustatory Nerves in the Dog.*

After the section of these nerves, the animal lost the tactile sensibility of the tongue, and retained the sense of taste; but, though it tasted, it appeared evidently to be by other parts of the mouth, and it seemed equally plain that it had lost the sense in the anterior part of the tongue, at least; for after it had been trained to suffer its mouth to be opened, quietly, and to have something laid upon its tongue, it allowed the pure coloquintida, moistened, to remain so long upon that part of it, care being taken to confine it to it, that no doubt could remain that the animal did not taste by the organ at its anterior part; but as soon as it was let go and closed its mouth, it displayed at once its sense of the taste by the ordinary indications.

### B. *Division of the Glosso-pharyngeal Nerves.*

This is difficult, and may be deceptive. The experimenter should dissect animals beforehand to know how to proceed—and he should dissect them afterwards, to be sure that he has proceeded right.

Dr. Alcock divided both glosso-pharyngeals in several dogs, with the following results: in two of them, each time that either nerve was taken upon a hook, preparatory to its division, a most violent action of the pharyngeal muscles was excited, resembling precisely the pharyngeal effort to reject the matter, which most persons must have experienced, when something nauseous or revolting has got beyond the power of the tongue, and entered the pharynx; and it was so imperative, that the animal struggled to get upon its feet, and was with difficulty restrained. In a third, a similar effect, only less marked in degree, was produced, by pulling upon the nerve with a ligature passed underneath it; and in one of the three, the root of the tongue was at the same time depressed, and rendered concave from side to side. In three of the animals, muscular startings occurred in the throat, while the nerve was held upon the hook; but the phenomenon just described did not, at that time, take place. In one of those the startings were strong and repeated; in the others, though obvious, they were less remarkable; and in one also, as has been mentioned, the first effect described was caused by pulling upon the nerve. In all but one, the animal displayed much suffering from stretching, pinching, and division of the nerve. In every instance deglutition was more or less impaired. In some cases deglutition was impossible. But under certain circumstances small moist morsels could be swallowed.

Taste did not appear to be much affected. The animal rejected food embittered with coloquintida, though it *appeared* to manifest less aversion than formerly. When the artificial circumstances under which animals are kept after operations is considered—this faint expression of doubt, induces us to think that taste was but little impaired.

C. *Division of the Branches of the Spheno-Palatine Ganglion.*

One of the branches of this ganglion is the vidian—other branches are distributed to the soft and hard palate, the tonsils, the gums, and Schneider's membrane.

Dr. Alcock has twice extirpated the ganglion from both sides in the dog. In both instances the animal tasted acutely.

In birds, who taste keenly, there is neither ganglion nor corda tympani discoverable.

D. *Division of the Gustatory and Palatine Nerves.*

The dog was favourable for this experiment, as an interval of some lines exists between the origin of the naso-palatine nerve, and the attachment to it of the extremity of the ganglion.

"I performed the experiment in two modes: in two dogs I simply cut the lingual branches below the jaw, and the palatines at the interval described, by which proceeding the influence of the ganglion over the latter was not interrupted; and in two others, at the same time that I cut the palatines, I also removed the ganglion; and in both cases the result was *quam proxime* the same, viz. that taste was very nearly obliterated, though sufficient evidence of its presence was still manifested to compel me to admit its existence. The effect produced was, that the animals, which had previously displayed the usual disgust at the coloquintida, no longer manifested any, but only ceased to eat food embittered with it: they would eat several bits in succession, and then refuse to take more, or drop the bit, taken, from the mouth, and take presently a clean bit if offered to them."

It must be well known to many of our readers that the naso-palatine nerve, is looked on by some as a branch of Meckel's ganglion, while others regard it, and the other nerves commonly considered branches of that ganglion, as really twigs of the superior maxillary division of the fifth, on which the ganglion, if it really *is* a ganglion, is placed.* Be that as it may, Dr. Alcock's experiments would appear not to be complete. He has divided the glosso-pharyngeals only—the linguals only—removed Meckel's ganglia only—and divided the linguals *and* the naso-palatine nerves between their origin and Meckel's ganglion. To render the deductions unexceptionable, he should have divided the latter nerves, the naso-palatine, per se. He would then have ascertained more exactly what such division would do.

The following are the conclusions drawn by Dr. Alcock:—

"In conclusion, I feel justified in

* Vide Swan, Cloquet, Arnold, and Cruveilhier passim.—*Eds.*

drawing the following inferences from the experiments and reasonings which have been detailed: 1st, that taste is a special sensation; 2d, that it enjoys two media of perception; 3d, that its media of perception are the glosso-pharyngeal nerves, and the lingual and palatine branches of the fifth nerves; 4th, that the glosso-pharyngeal nerves are not its special media; 5th, that the latter nerves both are sentient, and influence muscular action; and 6th, that the spheno-palatine ganglion and chorda tympani have no influence upon either the existence or perception of the sense."

Let us turn to Mr. Hilles.

His object is to render it probable, for he cannot prove it, that the use of Meckel's ganglion is to connect the senses of smell, sight, hearing, and taste, and to play the part of sympathetic in relation to them. That there is an association between those senses no one can deny—an association similar to that which obtains between the senses and the mind itself, nay, between the senses or the mind and many of the animal and vegetative functions. If, as Mr. Hilles says, the sight of a good dinner will make the mouth water, the *recollection* of it will do the same. If the sound of the uncorking of a bottle, awakes the thirst of the drunkard, will not other sounds occasion an emission of urine or of fæces? Does not Shakspeare tell us that—

Some men, when the bagpipe sings i'the nose,

Cannot contain their urine.

If an unpleasant piece of intelligence heard, excites the secretion of the lacrymal gland, will it not also arrest the functions of the stomach, and stimulate that of the kidneys? When Harry the Eighth bullied the Cardinal, he told him on their parting—

And now to breakfast with what appetite you may.

We might go on, but the matter is too trite. We do not intend to deny the sympathies which exist between the senses, but simply to observe, that they are but a section of a system of sympathies much wider, and less explicable by direct nervous communications.

Our knowledge of the ganglia, so far as it goes, does certainly tend to endow them with the power of combining the functions of the nerves attached to them, and of keeping up what we designate sympathy between them. What influence the ganglia exert upon nutrition it is difficult to say, yet analogy and probability imply that it is considerable. The nerves which are connected with Meckel's ganglia are apparently those of common sensation and of nutrition to the nares, the soft, and the hard palate. The Vidian nerve, pursuing a circuitous course, associates with the ganglion, the portio dura, the glosso-pharyngeal, the sympathetic, the gustatory, and, less directly, the hypoglossal. But, although it goes to the tympanum, we are not aware that it has been proved to have any direct communication with the acoustic nerve, nor have the naso-palatine branches of the same ganglion been proved to anastomose with the olfactory. But, on the whole, the conjectures of Mr. Hilles are probable, and agree with the received notions of the functions of ganglia of a similar character.

We hardly know what to say of the strictures of Mr. Noble on the experiments of Dr. Alcock. They breathe a degree of asperity which is disagreeable and out of place, and which contrasts with the urbane and scientific character of the observations of the latter.

The gist of Mr. Noble's arguments is the assertion, that taste is a special sensation, and *not* a modification of the common tactile sensibility of the tongue. He chiefly grounds this opinion on two cases. In the first, that of a patient labouring under one of the forms of facial paralysis, "the common sensation of one-half of the tongue was annihilated, while the sense of taste was unimpaired." In the second case, another of facial paralysis, he "considers" that his experiments rendered it clear that, "whilst the common sensibility of the left half of the tongue was but little impaired, its specific feeling was lost." Such are the two facts which Mr. Noble opposes to Dr. Alcock's experiments, and he opposes to them also a speculative consideration, that they are impugned by a recognized principle in physiology, "*that* a simple and special function must have but one simple nerve for its manifestation."

It requires no very subtle reasoner to perceive the fallacy of the last objection, and the weakness of the former. Why is it concluded that a nerve exists for a special sense? Because it is found to be the fact. Take the case of the eye. It possesses the power of vision, and its surface has a high degree of common sensibility. In the interior of the eye, where, according to the laws of optics, an image of objects should be formed, we find a nerve expanded to receive it, and transmit the sensation of the brain. If that nerve be destroyed, vision is lost, and reasoning, experiment, pathology, prove that it is the optic, the special nerve of sight. But on the surface, where sensation is, we discover branches of the fifth nerve, which is proved in many ways to confer that function.

Apply the same process of examination to the tongue. We find its structure exceedingly like those portions of the skin, where "touch" is most acute. We find that taste resides chiefly in that portion of the tongue, where touch is also best enjoyed. We find that to the same portion of the organ there goes one nerve, the gustatory, a branch of that fifth, which gives sensation to the surface of the eye, to the skin of the face, and to the nostrils and the palate. If the analogy of the special senses proves any thing, it proves against the notion that taste is a special sense—because, in their cases, there is obviously a special nerve for the sense, and a superadded one for common sensation, while, in the tongue, there is but *one* nerve in *that* part, where taste and touch are both most exquisite.*

The cases he cites are not conclusive when weighed against the results of direct experiment, *and* when balanced in the scale of severe inquiry. It must be difficult to determine the loss of taste, when only one half of the anterior part of the tongue is deprived of it. For the experiment to be accurate, the tongue must be exposed, while we know that, for taste, pressure, moisture, and associated actions are requisite to a greater or less degree.

If, too, as is most likely, the gustatory is a nerve both of touch and taste, it is not inconsistent with what we know of the nervous system to suppose, that one function may be more impaired than the other, in a palsied condition of the nerve.

On the whole, a consideration of the anatomy, and the study of the functions of the tongue, the palate and the fauces, when supported by experiments so careful, and, apparently, so unobjectionable, as are those of Dr. Alcock, will warrant, we conceive, the following inferences:—

1. *That* taste, in the common acceptation of the term, is probably a composite sense, made up of qualities appreciated by the tongue, the palate, the fauces, and even the nares.

2. *That* it is difficult to separate, in a satisfactory manner, the sapid and odorous qualities of bodies, or rather, it is difficult, to distinguish their appreciation.

3. *That* taste and common sensation appear to be allied, in their own intrinsic characters, in the nature of the anatomical structure adapted for their development, and in the nerves which form the media of perception of them.

4. *That* taste is most perfect in the anterior portion of the tongue, and most dependent on the gustatory.

5. *That* the palate and the dorsum of the tongue, are endowed by the palatine nerves, and by the glosso-pharyngeal, with a power which we cannot distinguish from taste, though it seems much inferior to that conferred by the gustatory. Indeed it may be doubted whether the glosso-pharyngeal does confer what may be considered taste at all.

6. *That* it is a subject of well founded

* We discard the hypoglossal. We cannot suppose that Mr. Noble can deem it a nerve of sensation, when it scarcely goes to the surface, where that lies.

regret, and not a little astonishment, that the experimenters on this subject, did not begin by ascertaining with something like precision, the respective powers of different parts of the mouth, in regard to taste, before they attempted to determine the quota contributed by different nerves.

---

### Emmenagogue Properties of the Hydriodate of Potash.

In a recent number of our Dublin contemporary, Dr. Pinching has drawn the attention of the profession to this remedy, which is now coming into very general use in various diseases.

The first patient on which the medicine was tried, was a female who had contracted venereal affections from her husband, and who had been repeatedly salivated. She had extensive phagedenic ulcers of the throat, which resisted sarsaparilla and other medicines. The hydriodate of potash was given in doses of ten grains three times a-day, with half an ounce of the tinctura camphoræ compos. daily, the bowels being regulated by castor oil. A good appetite was the first good effect of this plan, with refreshing sleep, and cleaning of the ulcers, which, in two months, disappeared. There had been no catamenia for four years previously. They now re-appeared, and continued regularly. Another case is detailed; but, as several remedies, besides the hydriodate, were employed, it is not so satisfactory.

---

### On Cerebral and Spinal Apoplexy, Paralysis, Convulsion, &c. of New Born Infants. By Dr. Kennedy.

[Dublin Journal.]

We agree with this talented practitioner, that the diseases of infants are not so easily recognized or diagnosed as authors aver them to be. The mere act of crying, or the expression of the features, are poor substitutes for reason and the powers of communicating the feelings in the sick. There is considerable difficulty in the treatment too, from the natural antipathy which infants exhibit to physic, and the bad effects of forcing them to take it, during which they are thrown into violent paroxysms of passion.

In respect to cerebro-spinal pathology our author acknowledges that we have only arrived at the threshold of the subject, as far as diagnosis is concerned. The causes of this disease in infants may, he thinks, be traced to the sudden change in the balance of the circulation and respiration at birth. The attack may be a simple primary affection, exhibiting all the symptoms of apoplexy.

*Case* 1. A child appeared well, up to the sixth day, when it refused the breast, and suddenly fell into a state of stupor, with stertorous breathing, &c. Two leeches were applied to the head, after which the pulse rose from 60 to 120, with natural respiration. It could not suck, however, for a day or two, when it recovered. The train of symptoms were apparently dependent on simple congestion of the brain.

Case 2.—*Apoplexy with tonic spasm.* This child had a tumor on the occiput, the effect of pressure in labour. In a few hours after birth it became insensible, with laborious respiration and spastic contractions of the muscles of the neck and lower extremities resembling opisthotonos. The mæconium was freely evacuated by castor oil—a leech was applied to the fontanelle, and two to the spine—warm bath—calomel in small doses—stimulating frictions to the spine—turpentine injection. The child recovered.

Case 3.—*Secondary Apoplexy.* A child who had presented symptoms of biliary derangement and yellow skin from birth, fell into stupor and stertor on the sixth day with contracted pupils. In six hours general convulsions ensued. He recovered by nearly the same means as those already detailed. It is supposed that the apoplexy was here consequent on the biliary derangement.

*Case* 4. In this case apoplexy appears to have been brought on by leaving the child on his face for some hours. He was taken up apoplectic, the inspirations convulsive, the expirations tardy—heart acting slowly. Various means were used, but without success, and he died in six hours. The veins on the surface of the brain were found to be turgid, and blood oozed out freely when the brain was sliced. There was half an ounce of fluid in the ventricles—cerebellum congested—spinal veins turgid—lungs little crepitant, and filled with black blood—larynx filled with reddened mucus.

### SPINAL APOPLEXY.

We shall abbreviate one or two cases from this division of the paper.

*Case* 5. A child died in 30 hours of trismus nascentium. On examination a considerable quantity of blood was found effused outside of the theca, and the veins of the medulla spinalis were very turgid and black. This case coincides with one related by Dr. Abercrombie, where blood was found effused in the spinal canal, exterior to the theca.

*Case* 6. On the fifth day from birth a child was suddenly seized with screaming, which continued for an hour. It then fell into a long and profound sleep, bordering on stupor. Next morning, the face was livid, eyes shut, mouth drawn to one side, arms fixed to the sides, breathing hurried, abdomen tense. It died in twenty hours. The brain, abdomen and thoracic viscera were healthy; but there was remarkable vascularity and turgescence of the spinal and medullary vessels. This might be denominated plethora spinalis.

*Case* 7. A healthy boy was attacked on the third day from birth, with a general convulsive paroxysm, after which the arms remained fixed, and the face livid. Leeches were applied, and aperients given, but without success. He died in twenty-eight hours.

On dissection the brain was found much congested, while the vessels of the medulla spinalis, the thecal vessels, and those about the origins of the nerves, were extremely distended. There were spastic contractions of the intestines, and the secretions were morbid in this case.

Paralysis is not very unfrequently met with in new-born infants—occurring either from injury to the nerve in the part paralyzed, or in the course of the nerve after leaving the head or spine.

*Case* 8. A woman had a tedious labour, the head of the child remaining long in the pelvis low down. On birth a tumor was observed on the scalp, together with a sloughing spot on the left parietal bone. The next day there was a remarkable alteration in the child's countenance—the angle of the mouth being drawn to the right side, even when the child was quiet, but very much so, when the child cried, attended by general distortion of the features. In other respects the child was healthy, and was taken from the hospital in the above state. Two or three cases of an analogous nature are detailed, but we need not notice them here.

*Case* 9. This was a case of hemiplegia of the left side, with ptosis, and paralysis of the portio-dura of the right side. Immediately after birth a soft tumor was observed on the right side of the head and vertex, with some excoriations on the left. The left eye was closed—the mouth drawn to the left side—distortion of the face when crying— hemiplegia of the left side of the body. The child recovered, after the application of a leech, warm bath, and stimulating liniments.

### CONVULSIONS OF THE NEW-BORN.

These affections are the most common of all. The late Dr. John Clarke maintained that, in all such cases, the brain was organically affected, either directly or indirectly; whilst Mr. North denies this position. The truth seems to lie between the extremes of opinion. That convulsions can take place without irritation of the brain or spinal marrow at the moment, few practitioners will now aver. But the nervous centres may be irritated without being inflamed or changed in structure. Convulsions are generally symptomatic of irritation in some other part than the brain, though the latter must be engaged in the morbid circle of association, before the development of convulsions. Dr. Kennedy adduces several examples of infantile convulsions—one where catalepsy supervened on convulsive action of the muscles of deglutition—and where the child was taken from the hospital in a cataleptic state—one of convulsions preceding miliary eruption—and two fatal cases, where apoplectic symptoms ushered in death, and where the usual post-mortem phenomena were found.

We have thus analyzed the paper of Dr. Kennedy, which is of a practical nature, and contains useful facts, without any pretension to great novelty or originality.

---

### Contagious Typhus.

Dr. Perry, one of the physicians to the Glasgow Infirmary, and a man of observation and candour, has published some remarks on this subject in the Dublin Journal. His paper is in the shape of a criticism on that of Dr. Lombard, of Geneva, which will be found in our present number. We need not enter on the points discussed, but confine ourselves to stating the results of the personal observation of Dr. Perry.

He had, for some years, entertained the opinion, founded upon an extensive series of observations, that contagious typhus is an *exanthematous disease*, and is subject to all the laws of the other exanthemata; that, as a general rule, it is only taken once in a life-time, and that a second attack of typhus does not occur more frequently than a second attack of small-pox, and, judging from his own experience, less frequently than a second attack of measles or scarlet fever. He ridicules the idea that contagious typhus may originate spontaneously from filth, bad diet, crowding, &c., and terms such notions "fancies, contrary to sound philosophy." There is certainly some plausible evidence extant, in support of opinions of the nature that he scouts, but Dr. Perry openly professes his utter want of faith. Yet he is not so sceptical as might be expected upon other points. He is satisfied, for example, that typhus fever is not contagious before the ninth day, or perhaps not till a later period than that. He thus states the reasons for this supposition.

"Among many circumstances which establish this opinion, I may mention one experiment which I made upon a pretty extensive scale. The fever wards of the Glasgow Royal Infirmary are each capable of containing twenty patients. The beds are arranged in two opposite rows, and are pretty near each other. While the patients are in the acute wards, they are not allowed the use of their clothes, though they may be able to set up; they are, therefore, almost constantly confined to bed, excepting when rising to stool; and there is about one close-stool to every three patients. Into the fever-house are admitted, cases of measles, scarlet fever, and small-pox; and patients are very frequently sent in, labouring under bronchitis, pneumonia, erysipelas, and other local inflammatory affections. I found by experience, that when the latter class of patients were sent to the convalescent ward, where they necessarily mixed with the others, almost all those who had not a previous attack of typhus fever were either seized with it before leaving the house, or returned soon after their dismissal, labouring under it; the period intervening between the time of their being sent to the convalescent ward, and the attack, never being less than eight days. Although means were taken to keep those recovering from small-pox, scarlatina, &c. in a separate room from those convalescent from typhus, the rooms being adjoining, the non-intercourse was incomplete, and the result was, that these diseases occasionally spread among the typhous convalescents, and the convalescents from small-pox and scarlatina caught typhus. In consequence of these observations, I adopted the practice of not sending, as formerly, to the convalescent wards, those patients affected with inflammatory diseases, unless I ascertained that they were secured against the disease by having had a previous attack of typhus; but kept them in the acute fever wards till they were so far recovered as to go to their own houses, and the result was, (and the practice was continued for several months,) that not one of those detained in the acute wards caught the disease while there, or returned with it afterwards. From the above, and other observations, I have adopted the opinion, that typhus, like measles, small-pox, &c. is chiefly spread during the period of convalescence."

Dr. Perry recommends the universal adoption of the practice of shaving the head of every fever patient admitted into the Glasgow Infirmary, and of washing well in a warm-bath every such patient, when dismissed. We are gratified to learn that he is now preparing statistical tables to illustrate points connected with fever. Its prevalence in Glasgow, and his situation at the Infirmary, will render such tables at his hands very valuable.

---

### Ulceration of the Brain.*

This is so rare as to confer a degree of interest on any well-authenticated case of it. Dr. Hannay, a zealous professor of Glasgow, has published the following in our contemporary of Dublin.

*Case.* A. G. æt. 9, Feb. 22, 1829, active, thin, and of unhealthy aspect.

Has violent pain in the forehead and heat of scalp; is pale, downcast and oppressed; pains dart suddenly through his head from ear to ear, and make him scream; he has had a most distressing night from this cause. He always applies his left hand to his brow. Nothing particular observable about his eyes; sickness and vomiting. Febrile heat and rapid pulse, 116; bowels open; tongue furred; thirst. Has for some time past been very "cold-rife," creeping close to the fire, and last night he had a slight rigor. He has been attending school regularly till three days ago. When an infant he received a severe blow on the occiput, but it has not troubled him since; and no external marks of the injury are apparent. He has long laboured under a severe cough, and occasional pain of chest, which followed an attack of pertussis. Leeches and a solution of tartar-emetic were the principal measures employed. In the night of the 23d, the pain grew so severe that his friends gave him ten drops of laudanum. He slept after this, and next morning had no pain; but there were languor, sighing, and oppression, and he complained that he saw red streaks on the wall in the dark. Pulse 68—tongue clean and moist. The leeches were repeated, and a blister ordered, but the friends refused to put it on. On the 24th, the pain in the head was severe, and there was delirium; pulse still slow. On the 26th, the tongue was furred, and the pulse is *reported* 28. Calomel and opium were the remedies now in use.

27th. Complains of much pain in his head, and of noises as if a piano were jingling in the room; great distress; pulse wiry, quick, and regular; eyes still unaffected; delirious occasionally; thirst; heat of surface; pain of head aggravated by motion. On opening the jugular vein on each side, no blood flowed. From the temporal artery eight ounces were got very quickly. The pulse reduced in force but not in frequency. Says pain of head is less, pale, and lies quite exhausted; pupil of right eye is irregular, and dilated on the light of a candle being cast on it; the left eye is natural; pain now referred exclusively to the left side of the head. Both previous blisters have risen badly; no stool; no vomiting.

At 3, p. m. of the 28th, he died.

*Dissection.* The sinuses of the brain, and the veins of the membranes, were distended with blood. There was a small quantity of serum between the

---

* Dublin Journal, Jan. 1837.

dura mater and the arachnoid, which was opaque. The pia mater was very vascular. One or two small patches of coagulable lymph were seen on the pia mater; one on the left lobe of the cerebellum, under the tentorium; another on the under surface of the left middle lobe. They presented a ramified appearance, being deposited in the course of the large vessels. The membrane at these places appeared thickened, and the substance of the brain immediately underneath felt decidedly softer, more pulpy than at any other part, but, with these exceptions, it had a natural degree of firmness. The surface of a section of the brain presented a more than ordinary number of bloody points.

On the surface of the left anterior lobe, on its outer and under side, there was a cavity or excavation of the convolutions, partly smooth, partly granulated, of a pale primrose hue on some parts, and of the cineritious colour of the brain in others. The granulations were small, whitish, shining bodies, and gave to the surface of the cavity the appearance of being sprinkled over with oatmeal in a moist state. Over part of the surface there was a cobweb-like expansion of small vessels: this was principally on the scabrous or granulated part. The cavity was of an irregular oval shape, with an area greater than that of a shilling; its edges were more than an eighth of an inch in depth, and fringed with the membranes which had been destroyed by the ulceration; its surface was as soft as thin paste or starch, but not diffluent. The corpus callosum, fornix, corpora striata, thalami, and cerebellum, exhibited no traces of disorganization, and except the turgid state of their vessels, appeared to be healthy.

The lungs were universally tuberculated. Some of the tubercles were miliary. Others were consolidated, and approaching the softened state.

The mucous membrane, of the intestines, we presume, presented, in many parts, an ecchymosed or injected appearance.

Dr. Hannay candidly owns that he did not suspect, during the boy's lifetime, the existence of ulceration of the brain. The appearances observed by Dr. H. correspond with those described by Dr. Craigie, as characteristic of the alteration; indeed its actual nature cannot be mistaken or denied.

"The records of cases of this nature do not present us with any pathognomonic feature, any symptom by which we can recognize its occurrence. Indeed, I do not find that its existence was anticipated in any one of the cases of which we have descriptions. It is worthy of remark, that there were no convulsions, no rigid contraction of the flexor muscles, nor paralysis, though these were prominent symptoms in most of the cases detailed to us. In having an inflamed state of the pia mater, and in the brain being softened at other points, it corresponds with the cases reported by other observers, Haller, Stall, Scoutteten.

In the two cases described by Scoutteten, there was severe disease of the alimentary canal; and in the case before us, indications of gastric derangement early presented themselves, and formed an urgent part of the sufferings; distinct and extensive traces of gastro-enteritis appeared on inspection.

Scoutteten is of opinion that, in the cases he saw, the inflammatory affection of the brain and membranes was subordinate to the phlegmasia of the alimentary mucous membrane. The case given by Morgagni presented severe abdominal disease.

The intermission of pain which occurred in this case has, I find, been noticed by others in cases of ulceration of the brain. And in one case recorded by Scoutteten, pain of head did not exist."

---

### Circumscribed False Aneurysm of the Brachial Artery, caused by Puncture in Blood-letting, and cured by Operation.

Cases of this description are possessed of interest to the practical surgeon. It has lately been proved, satisfactorily enough, that when the brachial artery, or a large branch of it, in the case of "high division," is wounded, pressure judiciously applied is very frequently successful. This fact is calculated to allay the apprehensions which have too often and too long infested the minds of the profession, and deterred them from remedying promptly and safely, the effects of an accident in venesection.

But when aneurysm has once occurred, an operation is generally necessary. A case of this kind has been communicated by Mr. Wall, resident surgeon to the Cork General Dispensary, to our valued contemporary of Dublin.

*Case.* Patrick Connell, aged 27, was admitted into the dispensary, on the 11th of August, 1836, labouring under head-ache, and affected with a circumscribed pulsating tumor, of the size of a pigeon's egg, at the bend of the arm. It was an aneurysm of the brachial artery, and had followed venesection performed in the median basilic vein, five weeks previously.

Mr. W. tried pressure, combined with antiphlogistic measures. It induced inflammation of the skin, and was abandoned in eight days. He tied the brachial artery above the tumor, on the 27th. The size of the latter immediately diminished. The pulse was discovered in the radial artery on the 3d of September. The ligature separated from the brachial on the 12th. On the 3d of October the wound was healed, and the aneurysmal tumor had disappeared. The only facts we think it necessary to particularize, relate to the temperature of the two limbs, after the performance of the operation.

*Evening of the day of operation.* Temperature of the affected limb in axilla 98; bend of arm 92; palm 90. Temperature of the sound limb, in axilla 96; bend of arm 97; palm 98. Pulse at wrist of left arm 84.

*First day after operation.* Temperature at axilla 99; bend 96; palm 92. Temperature of left arm at axilla 99; bend 96; palm 96.

*Second day.* Temperature at axilla 94—bend 89—palm 92.

*Third day.* Temperature at axilla 93; bend 92; palm 93; in the palm, under bandage, 94.

*Fourth day.* Temperature at axilla 96—palm 92.

---

MURIATE OF BARYTES FOR WHITE SWELLINGS.

The following observations contained in our Dublin contemporary, and extracted from the Gazette Médicale, are worth noticing. It appears that M. Pirondi, of Marseilles, has strongly recommended the use of muriate of barytes in cases of white-swelling, and that M. Lisfranc has adopted and countenanced the remedy. The observations we proceed to quote are taken from M. Lisfranc's Clinique.

Six grains of muriate of barytes are dissolved in four ounces of distilled water, of which one spoonful is taken every hour, except one hour before and two hours after each meal. In order to tolerate the medicine, the patient must abstain from wine and meat, taking only water and vegetable food. The bottle should not be exposed to the sun, or the salt will be precipitated, and the last spoonfuls contain a greater quantity; to avoid this, it should always be shaken. Sometimes the medicine produces slight pain in the stomach, or a feeling of weight; but if other symptoms do not follow, the stomach gradually becomes accustomed to the remedy, and the pain ceases. If, on the other hand, nausea, vomiting, or even some slight symptoms of poisoning come on, the medicine should be suspended, and cautiously resumed. The climate has some influence; for, although at Marseilles two drachms have been given, M. Lisfranc has never been able to increase the dose in Paris beyond forty-eight grains, and often he has been unable to reach that. The unpleasant symptoms have been removed by whites of eggs. Numerous patients have been submitted to this treatment, and the following are the conclusions which M. Lisfranc has arrived at.

1. Generally the white swelling has been much amended, and sometimes cured. 2. The benefit has been greatest amongst the scrofulous. 3. In some very few cases the muriate alone has cured. 4. After a certain time, the disease having become stationary, it was necessary to employ another method. At a later period, the renewed use of the muriate has produced excellent effects. 5. It may be employed both in the acute and chronic stage of white swellings. 6. Serious accidents have never resulted from its use; the slight symptoms before-mentioned have always yielded readily. 7. A frequent effect is a diminution in the frequency of the pulse; this falling from sixty or eighty to forty or fifty, or even to twenty-five. 8. In some circumstances the medicine, continued at the dose of twelve grains during the month, has produced as much amendment as in other cases where the dose has been gradually augmented. 9. Where the patients have been slightly inconvenienced with the medicine, it has been most useful. 10. Compression and local abstractions of blood have been often combined with this treatment, and with extreme advantage.

M. Lisfranc considers muriate of barytes, given according to M. Pirondi's method, as a truly valuable acquisition to surgery, ("une vraie conquête chirurgicale.")—*Gazette Médicale de Paris*, No. 14. 2 Avril, 1836.

Observations of this sort are well calculated to disabuse those, who look to the precepts and practice of continental medicine and surgery for improvement in their own. They are excellently adapted to display the folly of turning from the stores of facts we have at home, and from the sober experience of our countrymen, to France or Germany for practical instruction.

In the first place we see a most eminent Parisian surgeon, so ignorant of the improvements we have made in the pathology and treatment of diseases of the joints, that he not only makes use of the term "white-swelling"—a term which means any thing or nothing—a term which even the old women in this country have given up—but he evidently lumps under it a variety of diseases quite different in their characters, causes, and results.

In the second place, he recommends one internal remedy for such various, nay, such opposite complaints, and most preposterously draws a sort of average of its effects. Suppose we call the scrofulous disease of the joints white swelling. What shall we think of using a medicine like muriate of barytes, combined with a diet of vegetables and water, for that complaint? Suppose we say that white swelling means ulceration of the cartilages. What can muriate of barytes, or any other internal remedy effect, *unless* conjoined with those local measures which are of such obvious importance? In short, it is clear that the whole of the experiments on the employment of the medicine are not worth one farthing, because we are left in ignorance of the precise nature of the disease, for which it was exhibited. It was applied to all sorts of cases, without any definite understanding on the part of the experimentalist, of their variety.

---

### Extraordinary Deformity of the Pelvis.*

A woman gave birth to five full-grown healthy children, was then attacked with malacosteon, became pregnant, required the Cæsarian section, and died of it. A cast of her pelvis was presented by Professor Nægelé, of Heidelberg, to Dr. Montgomery, of Dublin.

In the dry preparation the distance between the promontory of the sacrum and the ramus of the pubes, is scarcely a quarter of an inch, so that when covered by the soft parts, these points must have been in contact. The greatest antero-posterior diameter at either side is only an inch and a quarter, and during life could not have exceeded an inch; the sacrum is bent in so as to form a right angle, and the ilia are folded or doubled in upon themselves, like softened pasteboard. Professor N. considers it "the most perfect specimen of contraction of the kind which

* Dub. Journ. Jan. 1837.

has ever been made known to the profession."

---

### Mr. Rankin on Croton Oil in Spleen.

*To the Editors of the India Journal of Medical Science.*

Gentlemen,—Having observed Dr. Hobson' communication in the *London Medical Gazette* of the 13th July last, on the use of croton oil as a counter-irritant, I was induced, some months ago, to try its powers on an inveterate case of enlarged spleen then in the 38th N. I. Hospital. The mode of application and the appearances produced are well described in No. 38, of Johnson's *Med.-Chirurgical Review*, page 448.

My patient was a seapoy, who, after remaining in hospital for some time under the usual treatment, without benefit, had been indulged with six months leave of absence on sick certificate, but had returned, about a month before, without the slightest diminution of the diseased viscus. Under these circumstances the oil was applied by friction over the diseased part, but whether from its inferiority, or from some peculiarity—from 15 to 20 drops were found necessary (at each application) to produce the desired effect. The patient complained a good deal, and the suppuration was very considerable, but within a week from its first application the spleen was perceptibly diminished, and the patient himself described it as feeling much lighter. At the end of three weeks the viscus was scarcely larger than natural, and the ulcerated surface being healed, he was discharged declaring himself quite well. I must not omit to mention that in treating the above case the Tinct. Fer. Mur. combined with bitter infusions, was administered internally—occasionally changed for the Sulph. of Quinine. But though perfectly willing to allow these remedies their full share of merit, the previous treatment amply proved that, even with the assistance of the Ung. Tart. Ant. externally, they were not adequate to the cure.

After treating several cases of less importance with similar results, I mentioned my success to Messrs. Barker and Row of the 66th and 73d Regt. N. I.—and was happy to hear, two days ago, that they had been equally fortunate. Mr. Row, however, found the oil prepared by himself, much more efficient than that furnished for the use of the hospital—its purgative effects being more conspicuous.

The frequent occurrence of visceral enlargement as a consequence of the fevers of the country, but I believe more particularly of Bengal, will probably render the above memorandum interesting, and I see no reason why the same remedy (with a judicious modification of the internal treatment) might not be employed with a prospect of equal success in cases of diseased liver, although I have hitherto had no opportunity of making the trial—that it may be used with *greater* benefit in many cases where the Tart. Emet. Oint. has heretofore been applied, I have experienced in several cases.

*Mynpoorie, Sept.* 29, 1834.

---

### Pemphigus Simplex.*

A case of this unfrequent complaint is related. It occurred in a healthy looking young woman, æt. 26, just arrived from England, and nursing.

She has a vesicular eruption over the face and neck, and extremities. It consists of distinct bullæ of various sizes, from that of a pea to that of an egg, containing a limpid transparent fluid without inflamed or elevated base; in other parts there are small erythematous patches slightly elevated, and brightly red; this appearance precedes the formation of the vesicle with much itching, the bullæ when fully developed, burst and discharge their fluid contents, then either dry off in a scurf, or form a thin scab, which falls off without leaving any mark. There has been

* India Jour. March 1, 1836.

little or no fever; the tongue is clean and moist. Bowels confined.

Castor oil with a drink of cream of tartar and antimony were the means employed. On the 3rd day, we find the following report.

Several small vesicles inside of the cheek and on the tongue. Eyes are turgid, the eruption is more general on the chest and shoulders, disappearing from the extremities; new vesicles succeed the disappearance of old ones, but the intermediate skin is of a natural appearance, and the limbs are not swollen.

In the evening of the 6th she had a slight shivering fit, succeeded by pyrexia, for which calomel and antimony, with purgatives, were ordered.

On the 10th day there was no fever, fewer vesicles appeared, and the ankle was slightly swollen. She was ordered quinine, with rhubarb pills, and on the 18th day, the eruption had entirely disappeared, and left only a trifling discolouration where the largest bullæ had been.

---

### Retention of Urine after Amputation of the Penis.*

A Fakeer either amputated his penis himself from devotion, or had it amputated by others for the reverse. It was removed close to the pubes. Gradual contraction of the urethra at the stump occurred, and, his case baffling the village doctors, he applied to Mr. Stuart, military surgeon, at Baroda. The patient was feverish, and had pain and irritation about the anus and the hypogastrium.

"The wound left by the removal of the penis was healed, and presented an irregular knobby surface, without any *perceptible* opening for the urine to escape. On desiring him to void his urine, he pressed with one hand above the pubis, and drew the other along the perineum, forcing out, with each stroke with his hand, the least possible quantity of urine. In this way he had relieved himself for several days. By watching narrowly I was able to discover a very small opening through which the urine oozed, and attempted to introduce a fine probe, but only succeeded to the extent of about one-third of an inch. I ordered him castor oil, and warm fomentations, directing him at the same time to continue his efforts in voiding as much urine as possible till morning, when something would be done for him. The following morning I saw him in company with Assistant Surgeon Stowell, he was less excited and feverish, but complained of great pain when pressed on the abdomen. It being impossible to ascertain the extent of the stricture (the flow of urine not being perceptible in the perineum) we determined on cutting down on the urethra as low as possible, and be guided by circumstances in completing the operation. The patient being placed as in the operation for stone, I commenced by making an incision along the raphé, through the skin, and cellular substance. On cutting through the ejaculator and corpus spongiosum into the urethra, an inch below the scrotum, it was thickened, and *apparently* impervious. The incisions were continued down to the bulb and a few drops of urine flowed. I then introduced a probe and lengthened the incisions till a full-sized catheter easily passed into the bladder. Upwards of a pint of ropy urine was discharged. The catheter was left in the bladder and the wound lightly dressed with lint dipt in oil."

All went on well, the wound soon healed, and he was directed to introduce a bougie occasionally. He could retain the urine perfectly. He subsequently lost his bougies, and contraction of the new orifice occurred. By dilatation this was again remedied. The case displays the tendency to contraction on the part of the urethra when divided, and the necessity of habitually employing bougies when it exists. It is a plain illustration of an important fact.

---

* India Journal, No. XII.

### Change of Form in the Head, concurrently with Change of Character and Talents.*

We must carefully separate the principles of phrenology from the details. Yet they are constantly confounded, especially by those who affect to laugh at the whole doctrine. If we grant, and it is difficult to disprove it, that the brain is the organ of the mind—and if we admit, which it is difficult to dispute, that what we term the mind is an aggregate of many qualities, it follows logically, that the material organ must itself be made up of many parts, adapted for those many functions. Absurdity does not stick to such a view, but to the converse, and, so far as reasoning can go, it points out not only its propriety, but its necessity.

When we leave this essential principle of phrenology, and endeavour to determine and verify its details, it must be owned that we meet with many difficulties. In the first place, we have to determine what the primitive faculties of the mind are—a metaphysical and abstruse inquiry. Supposing we succeed in that, a task, perhaps, more arduous still awaits us. We have to ascertain what portions of the brain correspond to the faculties we have agreed on. This investigation is no longer metaphysical, but one of strict observation. It would be out of place here, to enumerate the fallacies to which such observation is exposed; but it may be granted, by the most enthusiastic lover of phrenology, that when all that elevates his science above the mere enunciation of an abstract principle hinges on the accuracy of his facts, he is interested in securing such accuracy by every means in his power. Loose observations have damaged phrenology much —inferior observers have degraded it—mountebank professors of it have gone far to render it ridiculous. We have, on several occasions, stepped forward to impress on the minds of the really able phrenologists, the necessity of repressing the *niaiseries* of the small fry about them, a duty which they owe to their own reputations. We shall continue to point out now and then what appear to us to be vague or inaccurate statements.

Mr. Silas Jones, an American phrenologist, has published a work, for the purpose of shewing that great changes in mental faculties may be combined with corresponding changes in the form or dimensions of the cranium. This is a proposition which carries with it nothing improbable or ridiculous. But some of the facts that he adduces in its favour are not such as would convince the sceptical. For instance:

"I have several facts, founded not upon observations made from comparison of casts, but still they are such as to be entitled to our confidence. A young artist of my acquaintance had formerly been a dealer in dry goods, and a few years since commenced the business of portrait-painting. He had been absent for several years from his mother; when on a visit to her, she called him up to her, and observing every part of his countenance carefully, said, 'your forehead has altered in form since I saw you, all the lower part of it seems to be pushed out.' This was the careful observation of a fond mother, when tracing out the lineaments of a beloved son. It was no doubt true. Nearly all the perceptive organs are now very decidedly large; and he says they have increased in size since he commenced his new vocation. Young men in cities, it will be found, have greater power and activity in the perceptive organs, than those who have always been in country situations. There is a constantly changing succession of objects in cities, which give ample scope and stimulus to these organs. These rapid changes are unfavourable to quiet reflection, hence the knowing organs acquire a great ascendancy.

I have noticed in very many instances that experienced navigators have the organs of Locality very prominent, and probably in consequence of great exercise of them. So with blind people, these organs become very large. It is the case of a blind man in Boston, who

* Phrenological Journal, Dec. 1st, 1836.

travels in every part of that city without a guide."

We fear that the good old lady's astonishment at the growth of her son's forehead will not be deemed conclusive by the antiphrenologists, who may reasonably demand some more positive and accurate admeasurements than have been offered.

---

### Hunteriana.*

The following short anecdotes of some celebrated men in our profession, are extracted from the first volume of the complete works of John Hunter, edited, and just published, by Mr. Palmer. That volume will be noticed more fully in another place. In this, we shall confine ourselves to extracting a few anecdotes, which cannot but interest a profession which owes so much to the men whom they refer to.

It is not, however, the view of merely gratifying curiosity which prompts us to do this. We do not profess to cater for the amusement of our readers, but to lay before them what will tend to their advantage. Utility is our avowed and constant object; but utility in a comprehensive sense. The accumulation of facts, their analysis, comparison, are the means best calculated for attaining the end we have proposed. But surely it is useful to see what the ablest men have done, to mark the steps that they have trod upon the painful road to eminence, and to learn the indubitable truth, that success in their case, as in that of all, has been the result of unceasing and well-directed exertion.

There is a clique in the profession, who, hopeless of honest fame and of true eminence, are naturally jealous of those who have had, who have, or who are like to have, what, unwillingly, they are compelled to despair of getting. To their jaundiced eyes, all is indeed yellow. Their bile overflows at the mention of successful talent, and they vomit it in the shape of generous declamation against monopoly, aristocracy, and those thousand sins which, in spite of them, society *will* cherish. It is said that "there is a joy in woe." There must certainly be a pleasure in the expression of malicious discontent, independently even of that reward so sweet to ultra-liberals, we mean, "what it will bring." There must be such a pleasure, or we should not witness, so frequently as we do, the ostentatious and gratuitous exhibition of malignity. To these unhappy persons, the following anecdotes will be gall and wormwood, because the actors succeeded in life. In the right thinking part of the profession, the vast majority of its members, they will give birth to much delightful, and some useful reflection.

1. *Talent in the Families of the Hunters and the Baillies.*

"Mr. Wardrop, in his interesting life of Dr. Baillie, observes, that 'the extent of talent united in his family and connexions was remarkable. He was not only the son of an able Professor, and nephew of the two Hunters, but his sister, Miss Joanna Baillie, has attained the most elevated rank in literature. Mrs. Baillie's sister was married to the late Sir Richard Croft, a man whose name is endeared in the recollection of many, as well for his manly and upright heart as for his professional celebrity: and Mr. Denman, who has distinguished himself so much at the bar (now Lord Chief Justice), was Dr. Baillie's brother-in-law."

2. *What Lectures on Anatomy and Surgery were.*

It has lately been proposed by some, who profess to wish for a radical reformation of the profession, that the vile system of lecturing and of certificates should be burked, and that we should return to the practice of the good old times, when every master, an admirable Crichton, instructed his apprentice in every branch of medicine. It is easy to conceive the benefits that must spring from such a system. As it will, probably, be soon adopted, we may mention the mode in which the present infamous as well as ridiculous plan took root.

"It was customary for the lecturers

* The Works of John Hunter, F.R.S. with Notes. Edited by J. F. Palmer, Esq. &c. Vol. I. 1835.

of that day, 1745, to treat in one course on a number of subjects, sufficient to furnish matter for three or four distinct courses according to our present system; and the meagre amount of information afforded to their hearers may be judged of by the following facts, mentioned by Mr. Chevalier in his Hunterian Oration. Mr. Bromfield, who was surgeon to St. George's, and a lecturer of considerable note, comprised anatomy and surgery in a course of thirty-six lectures. Dr. Nicholls, at whose school Wm. Hunter studied, professed to teach anatomy, physiology, and the general principles of pathology and midwifery in thirty-nine, and Mr. Nourse of St. Bartholomew's embraced 'totam rem anatomicam' in twenty-three lectures."

This plan was an approach to perfection, for the next best thing to no lectures at all, must be lectures so curtailed as to be good for little. We have arrived at a formidable pitch of corruption. Now *one course* of anatomical lectures lasts for seven months; and comprises about 150 lectures, and as many demonstrations. A frightful enormity.

It is worth while observing the encroachments of the surgeons on the physicians. The former anciently possessed the learning and science of the profession—the latter were the handicraftsmen, and were kept down by their association with the barbers. It is customary to imagine that the divorce of the two professions of shaving and of surgery, was the cause of the latter. It was essentially its consequence. Of course it was advantageous to get rid of the pole and the soap-dish, but the progress of society, and the advancing education of the surgeons demanded it. The same circumstances operated to make the physicians really physicians, and to force them to abandon lecturing on anatomy.

"It was not until 1745 that the alliance between barbers and surgeons was happily dissolved: before this time, any surgeon dissecting a body out of their Hall was liable to a fine of ten pounds. Amongst other privileges, they possessed the right of claiming annually the bodies of four executed felons, which probably led Dr. Caius to choose the Barber-Surgeon's Hall, to deliver his anatomical lectures in, soon after their incorporation in 1540. From this time to the dissolution of the company, their readers of anatomy were, with very few exceptions, chosen from the College of Physicians, to whom, with the exception of Cowper and Cheselden, is due the merit of supporting the fame of this country in anatomical pursuits, from the time of Caius to that of the Hunters. In the list of physicians who successively taught anatomy in England, we find the names of Caius, Harvey, Glisson, Mead, Willis, Lower, Wm. Hunter, and last, though not least, of Matthew Baillie. With Dr. Baillie ended the race of Physician-Anatomists, who with the exception of Dr. Wilson, ... lectures at the present day. This resignation of the professor's chair of anatomy on the part of the physicians, arose not however from any lack of able men to fill it, but from a conviction that the surgeons were now fully capable of instructing their own pupils, and with more practical effect."

Since this passage was written, Dr. Wilson has resigned the anatomical lecturer's chair, and this "last rose of Summer" is like—

"Its lovely companions, all faded and gone."

### *Difficulties of Genius.*

Lord Eldon, we believe, observed, that most of the Lord Chancellors come from the garret. Most of our greatest men have come from apartments not very much lower. There is nothing like an empty stomach, for stimulating men to work. The gastric juice is a very excellent fillip to an idle man. William Hunter's means were limited, when he started in London.

"In proof of this, Mr. Watson, formerly surgeon to the Westminster Hospital, and one of Wm. Hunter's earliest pupils, used to relate, that as they were walking home together after the introductory lecture, the latter, who carried a bag containing seventy guineas, which he had received for entrance fees, remarked, that this was a larger sum than he had ever before been possessed of. Linnæus gives a similar account of his own slender beginnings where he says, 'Exivi patriâ, triginti sex nummis aureis dives;' and it is related by Sir James Earle, that at Pott's death, a small box was found among his papers, containing a few pieces of money, not amounting to five pounds, being the whole which he ever received from the wreck of his father's fortune. Such anecdotes may serve to encourage those who, at the outset of their journey through life, chance to have their purses but slenderly furnished: numbers more might be found by a reference to the lives of eminent men in all professions, many of whom, though they have afterwards reaped a bountiful share of the favours of Fortune, were doubtless obliged, on their first starting in life, to have recourse to shifts quite as curious as those of Johnson's Irish friend, who in describing how a man may live respectably in London on thirty pounds a year, allots ten for the expenses of clothes, and provides that all visits are to be paid on *clean shirt days.*"

*John Hunter amongst the Gods.*

Most people are aware that John Hunter was an idle, if not debauched young man. But they may not be aware, where his tastes led him, nor the sort of associates which genius such as his delighted in. He would seem, like "Prince Hal," to have relished the society of the coarsest, indeed the most brutal of mankind.

"Here, as in graver matters, his ambition urged him to take the lead of his companions, amongst whom he went by the familiar title of 'Jack Hunter.' Nor was he always very nice in the choice of his associates, but sometimes sought entertainment in the coarse broad humour to be found amid the lower ranks of society. He was employed by his brother to cater for the dissecting-room, in the course of which employment he became a great favourite with that certainly not too respectable class of persons the resurrection men! and one of the amusements in which he took especial pleasure, was to mingle with the gods in the shilling gallery, for the purpose of assisting to damn the productions of unhappy authors, an office in which he is said to have displayed peculiar tact and vigour."

A series of fortunate accidents combined to draw this boisterous, rude, and vulgar young man to the study of a science which his genius was to revolutionize. To William Hunter the profession is undoubtedly indebted for the development of John; and their history naturally reminds us of what has been said of Dr. Beddoes—"if he was the discoverer of nothing else, he was the discoverer of Davy."

*John Hunter preserved from being made an Old Woman.*

In 1753, Hunter entered as a Gentleman Commoner, at St. Mary's Hall, Oxford. Whether his brother wished him to become a physician or an accoucheur is not quite certain, but the following is John's own account of the transaction.

"In speaking of this period of hislife some years afterwards to Sir Anthony Carlisle, then a student at the hospital, Hunter said: 'They wanted to make an old woman of me; or that I should stuff Latin and Greek at the University; but,' added he, significantly pressing his thumb-nail on the table, 'these schemes I cracked like so many vermin as they came before me.' It was fortunate, probably, that he decided as he did, for though his future progress was slow, the roughness of his manners would have been a still greater impediment to him in either of the other branches of the profession."

This contempt for learning is itself contemptible. But in Hunter's case it is palliated by the results, and by the reflection that in him it was not the expression of the barbarian pride of an illiterate man, but the outbreak of a genius essentially one of action.

*Encouragement given to Science by the English Government.*

Our Government is the most philosophical imaginable. Whigs and Tories have in these latter days exhibited no difference of opinion upon one point—the folly of patronising science. To it they apply with the most exemplary rigour, the economical principles of supply and demand. If science is wanted, the people will pay for it—if it is not, let it go to the dogs. They have, properly enough, drawn a broad distinction between what is useful and what is not. Science is the former, and its utility should be left to support it. But the services of some young gentlemen and ladies are not capable of being appreciated by the public, and a discerning Government has accordingly taken *their* reward into its own hands. Thus it has happened that the pension list has exhibited the names of many excellent individuals, but of very few scientific ones. The former, to be sure, have usually happened to be relatives, dependents, or friends of the party in power at the time. But that was accidental, and if merit existed so very near home, it would have been the more inexcusable to have neglected it.

Within these very few years our public men have been shamed into something like respect for science. It must be owned, to the honour of Sir Robert Peel, that he led the way in evincing a practical regard for it, and in bestowing a mite from the public purse upon men of talent in need of it. After many wry faces the Whig Government has been forced to follow his example, and though it has been done in the most ungracious manner possible, it has been done.* Let us hope that the time is approaching, when a great and wealthy nation, raised and supported by industry and commerce, will evince its gratitude to the arts and sciences by which that commerce has been materially enlarged, and which give to civilization many of its comforts and almost all its ornaments—let us hope that the day is not remote, when a people who will spurn the idea of pensioning the parasites or pimps of minister or court, would freely extend their liberality to philosophy; and encourage, as a great empire should, the pursuit of studies, beneficial in their consequences to humanity, but not possessed of such immediate and practical utility, as to afford a decent and honourable subsistence to the individuals who prosecute them. What has been the practice the following anecdote will prove.

"Dr. Hunter had, in the year 1765, in the most liberal way, proposed to the then Ministry to build a public theatre of anatomy, at an expense to himself of 7000*l.*, and to endow a professorship of anatomy in perpetuity, on condition that they would grant a piece of ground in the Mews as a site for the building. But Lord Grenville and his colleagues, with the apathy which the English Government has too often shown to the interests of science, declined the offer."

*Hunter an indifferent Lecturer.*

A very slight acquaintance with Hunter's works, would create the idea that he was not adapted to convey information to young men. The great requisites for a successful lecturer are methodical arrangement, simplicity, and clearness of description, and a certain amount of eloquence. If the lecturer has genius too, so much the better; but no genius, uncombined with these requisites, can qualify him to excel in the oral instruction of young men. Let us glance at Hunter in the surgical chair. His class never exceeded thirty.

"To a pupil (Sir Astley Cooper) who asked with surprise, whether he had not the year before stated an opinion on some point, directly at variance with one he had just put forth; he replied, 'Very likely I did; I hope I grow wiser every year:' and to the same purport he answered Professor Coleman (another of his pupils), who asked whether he had not written so and so; 'Never ask me what I have said, or what I have written; but if you will ask me what my present opinions are, I will tell you.' Occasionally, too, he would say to any of the pupils whom he saw taking notes, 'You had better not write down that observation, for very likely I shall think differently next year:' and on one occasion, after lecturing for a considerable time, he stopped short, raised his spectacles, and said, 'Gentlemen, I think

* We allude to the pension of Mr. Faraday.

you had better omit what I have been saying; the fact is, I had an idea when I wrote down this, but I have lost the train of thought connected with it, and I cannot now recall it.' Such a mode of lecturing was not likely to become popular; his class consequently never exceeded thirty, and not half that number derived much benefit by their attendance."

*Hunter not a mere Operator.*

It would be either idiotcy or imposture to contend that the power of operating well, is not a highly desirable natural gift to one who intends to be a consulting surgeon. But it is as absurd to pretend that that power should not be subservient to the higher intellectual qualities—that it should not be regulated by judgment, caution, and humanity. The mere operator is both a dangerous and a disgusting person, and none but the very weak and very ignorant can either admire or trust him. Fortunately operations are becoming daily less frequent, because the advance of science teaches us to prevent the necessity for their performance in many cases, and to refuse to perform them in others. It is certain that Hunter was not what is called a dashing operator. He did not carve his way to reputation on the living. He did not aspire to be a prince of butchers. His head, not his hands, made him what he was—the intellectual, not the handicraft head of his profession. Like Cheselden, he felt that operations are really the disgrace of surgery. His sensibilities were not so fine as Cheselden's, and in him the sentiment was rather the result of reflection than of deep feeling.

"In the opinion of Sir Astley Cooper, whose judgment on the subject must be considered as at least as likely to be correct as that of any man living, Hunter scarcely deserved to be considered a dexterous operator, certainly not an elegant one; but his large experience, and his extraordinary skill as an anatomist, almost always enabled him successfully to complete any operation he undertook: though slow, he was sure. Hunter does not seem, indeed, to have been ever ambitious of particular renown in this field. He used frequently to say, 'To perform an operation, is to mutilate a patient we cannot cure; it should therefore be considered as an acknowledgment of the imperfection of our art.'"

*Hunter's early rising and little Sleep.*

Hunter generally began working before six in the morning, dissected till nine, breakfasted, saw patients at his own house till twelve, dined punctually at four, drank little or no wine, slept for about an hour after dinner, and went to bed about one or two in the morning. The following anecdote will shew his habits in respect to early rising. It may be questioned whether the "diluculo surgere saluberrimum," would apply so much to his body as his mind.

"On his arrival in London, Mr. Thomas, in company with Mr. Nicol, by whom he was to be introduced, called on Hunter; they found him dressing. 'Well, young gentleman,' said Hunter, when the first ceremonies of introduction were over, 'so you are come to town to be a surgeon; And how long do you intend to stay?' 'One year,' was the reply. 'Then,' said he, 'I'll tell you what, that won't do; I've been here a great many years, and have worked hard too, and yet I don't know the principles of the art.' After some further conversation, Mr. T. was directed to call again in an hour, which he did, and accompanied Hunter to the hospital, where he said to him, after the business was over, 'Come to me to-morrow morning, young gentleman, and I will put you further in the way of things; come early in the morning, as soon after four as you can.' It was summer: Mr. Thomas kept the appointment, and found Hunter, at that early hour, busily engaged in dissecting beetles."

*His Infirmity of Temper.*

Perhaps few *faults* bring their own punishment more certainly than a bad and irritable temper does. Molière, in his play of the "Grondeur," hits off the miseries of an ill-tempered person to the life. The man who will neither have the door open nor shut, must be a discontented one. Hunter, a man of great genius, strong passions, and no education, naturally gave way to an irritability which his labours tended to increase, and found in age the evil consequences of the want of that moral culture and discipline, which in youth had not been enforced by others, and which the strength of his manhood led him to despise. He was not on good terms with the profession—he quarrelled with his brother—and died from the effects of an altercation with his colleagues. Labouring under angina pectoris, continually exposing himself to contradiction and to mortification by his overbearing disposition and his ungovernable temper, his last years must have been far from enviable, and the confession was extorted from him, that "his life was in the hands of any rascal who chose to annoy and teaze him."

His words were borne out by the event. On the 16th of October, 1793, Mr. Hunter went down to the board-room of St. George's Hospital, the scene of many former contests with his colleagues, and brought forward the petition of two young men, that they might be admitted pupils of the hospital, in opposition to some standing orders of the governors. In the course of his remarks, he was flatly contradicted. He immediately ceased speaking, hurried into an adjoining room, and fell lifeless into the arms of Dr. Robertson.

He died of angina pectoris, some fits of which he had previously laboured under. The muscular structure of the heart was pale and flabby—the coronary arteries were ossified—so were the mitral valves—the aorta was dilated, its inner surface studded with opaque spots, and its valves thickened.

Thus died John Hunter, a man of no education, great genius, and wonderful industry. Whether education would have spoilt that genius, and damped that industry, is a question which cannot now be solved, and which different persons may be inclined to answer in different ways. As an abstract proposition, it will not be doubted that education, which means improvement of the mind, cannot possibly be injurious to it. But certainly particular *modes* of education do interfere with particular trains of reflection, and, by occupying our time and engaging our tastes in the acquisition of what others have thought and done, they tend to cramp the spirit of originality. Perhaps it was fortunate that Hunter had not the species of education which was then to be obtained. He lived in a transition period, when science required revolutionizing, and his rude, unlettered genius, exclusively directed, by his ignorance of antiquity, to the study of existing objects, was calculated to overpower, by the mass of facts that it accumulated, the scholastic dogmas of the age. Such a revolution must and would have taken place, had no John Hunter existed. But we owe it to him, that England has assumed the position that she has, in elevating medicine to a science. In this country, money is so necessary to all, that men of talent and ambition devote themselves to those immediately practical pursuits, which lead directly to profit and wealth. Education tends to encourage this, because it naturally makes those who are endowed with it solicitous to mingle with the classes in which education and refinement obtain. A man like John Hunter would have no such feelings. His unacquaintance with literature unfitted him for polite society—his bad temper and worse manners shut him out of it—his feelings and his tastes pointed out for his associates those to whom he was not conscious of inferiority—to enjoy such acquaintance, and the pleasures they would bring, little wealth was requisite—and we cannot be surprised if the chum of resurrection-men, and the damner of plays in the one-shilling gallery, should feel that the pursuit of wealth was even irksome, in comparison with the undisturbed prosecution of his peculiar studies and amusements. When called to a patient while dissecting, he exclaimed to Mr. Lynn—"Well Lynn, I must go and earn this d——d guinea, or I shall want it to-morrow."

Untrained genius like Hunter's is ever in extremes. The cold rules of mediocrity and prudence are unable to restrain it, whichever way it points to, right or wrong. A first-rate burglar, were circumstances different, might be the raw material of a hero—and the greatest vagabonds are notoriously the most clever fellows of their class. The deer-stealer, who was subsequently the poet "of all time"—the associate of Falstaff and of Pistol, who proved one of the greatest of England's Kings—the debauched and riotous anatomical student, who was afterwards John Hunter, are all examples of this moral truism. The spectacle of his brother William Hunter acquiring fortune and fame—the emulation gradually excited by the success of men whom he felt to be in talent his inferiors—the consciousness, perhaps, to a noble mind so dreadful, of time lost, opportunities wasted, powers thrown away, all goaded him on to those wonderful exertions that he made, and developed that enthusiasm, the offspring of genius and the agent of its achievements. Such enthusiasm was exhibited in a remarkable degree by this extraordinary man. An instance or two will exhibit at once his love for science, and his disregard for pecuniary considerations.

While engaged in his researches upon whales, he engaged a surgeon at his own expense to proceed to the North in a Greenland whaler, after instructing him in anatomy, and furnishing him with all that was useful. All he got in return was a bit of whale's skin, with some barnacles stuck on it.

When O'Brien, the Irish giant, died, particular care was taken of the body. Mr. Hunter actually paid five hundred pounds to get possession of it.

Here we must pause. John Hunter is not only an individual, but the representative, of an age gone by. There is, and there always will be, an ample field for industry and talent. But their application must vary with the character of the times, and, in our own, we fear that the object they will point to, is wealth. Of Hunter we may safely say, that "we ne'er shall look upon his like again."

---

### Obscure Medullary Disease of the Stomach. By Jas. Johnson, M.D.

A gentleman (Mr. Sandy), aged about 45 years, became affected with uneasy sensations about the stomach or upper bowels, varying from an hour to three or four hours after taking food, especially the dinner meal. The pain was seldom acute, but rather it was a sense of distention, attended with eructations of air and acid, and continuing till the digestive process was finished. There was no vomiting or sickness—or scarcely any, during the whole period of the disease, which lasted 16 or 18 months. At an early period of the malady, and many months before he came under my care, he passed, as was supposed, a mass of hydatids. It is probable that this was a mistake, and that the discharge consisted of gelatinous matters, which occasionally passed afterwards, while I attended him. He had been attended by several medical gentlemen, none of whom could make up their minds as to the real nature of the disease, or even its precise seat. This was also the case with myself. When I first saw him in July, 1836, he was emaciated, sallow, and unhealthy in aspect. On stripping him there was no tumour visible or tangible in any part of the abdomen. But when he strained, there could be felt a very small induration three or four inches above and to the left of the umbilicus, devoid of pain on pressure. This induration could not always be felt at first—but for the last two or three months of his life I could always feel it, and considered that it had got larger.

The phenomena of pain after food still continued, as well as the emaciation, without any fever or other constitutional derangement. About the middle of November he was seized in the night with syncope, and he was supposed to be dying. A surgeon in the neighbourhood was called up, and administered some cordial, by which he was revived. I saw him the next day, and found him much paler and more depressed than he had been. On exmining the abdomen, there was a greater fulness in the epigastrium than usual, but the distinct induration could no longer be felt. This puzzled me a good deal. There was no motion for three or four days, although aperients were exhibited. At the expiration of this time a large substance was passed, after great difficulty, together with much fæces, and, on examination, there appeared in the commode what resembled exactly about eighteen or twenty inches of the ileum inverted and reddened. It could be lifted up on a stick and handled, without breaking to pieces. This created alarm, and I was summoned. I could not, at first, determine whether or not it was an organized substance. It was taken to the Anatomical School in Kinnerton Street, and there washed and examined carefully. It was found to be a coagulum of blood, deprived of much of its colouring matter, and so exactly taking the mould of the small intestine, that the valvulæ conniventes, &c. of the bowel were most beautifully exhibited. On examination of the abdomen now, the fulness of the epigastrium was gone, and the original hard knot was felt as usual. The bowels now became regular and healthy, as were all the secretions. But the pain and sense of distention, some hours after taking food, became gradually more and more distressing, so that, for some days, he could hardly be prevailed upon to take any thing but liquids. After the complete digestion of the food, he got free from all uneasiness, and was cheerful and comfortable till the next day after dinner. All this time there was no sickness or vomiting; but the flesh gradually and steadily vanished, till he became a mere skeleton. In December and January last, the pain and sense of distention after food became so great that he was obliged almost to starve himself to avoid the tortures of digestion. This, of course, accelerated the emaciation, and he died exhausted on the 13th January, 1837.

The body was examined next day by Mr. Henry James Johnson, and Mr. Hutchinson, of Farringdon Street—a gentleman who had formerly attended the patient. The following were the minutes of dissection.

"1. Body much emaciated.

2. No induration perceptible *after death*, through the abdominal parietes.

3. *Abdomen.* The stomach was concealed by the transverse arch of the colon, which overlaid it, and which, at its left extremity, was bound down by adhesions in the left hypochondrium.

The stomach was small. The left lobe of the liver was intimately united to it; the lesser omentum being almost obliterated.

On opening the stomach, its internal surface presented numerous projections of medullary structure, which extensively occupied the lesser, the greater curvatures, and the cardiac end of the stomach, as well as the termination of the œsophagus in the stomach. The mucous membrane covering the morbid growths was ulcerated, as were the morbid growths, in some degree, themselves.

The pylorus was healthy. The termination of the duodenum in the jejunum was adherent to the morbid growth of the stomach.

The intestines were healthy."

The can be little doubt that the hæmorrhage which formed the remarkable coagulum some weeks before this gentleman's death, proceeded from one of the ulcerations in the stomach—and there is great probability that the supposed cluster of hydatids discharged at an early period of the disease, was only some morbid secretions from the stomach or intestines. None of the medical gentlemen who saw this patient hit upon the true pathology, some considering it one thing and others another. I could not bring myself to think the stomach itself was the seat of the disease, from the absence of vomiting, and from the long interval between the taking of food and the commencement of pain. The adhesions between the stomach and left lobe of the liver, as also those which bound down the transverse arch of the colon that concealed the stomach, contributed much to the difficulty of diagnosis. The case, however, is very instructive, as shewing how accidental circumstances may render the diagnosis and prognosis most perplexing and erroneous.

